# Supplementary material for: Sub-second and ppm-level optical sensing of hydrogen using templated control of nano-hydride geometry and composition
Source: Nat Commun. 2021 Apr 23;12:2414. doi: 10.1038/s41467-021-22697-w (PMC8065102; doi:10.1038/s41467-021-22697-w)
Supplement: Supplementary file 1 — Supplementary Information [file 41467_2021_22697_MOESM1_ESM.pdf]

Supplementary Information

Sub-second and ppm-level Optical Sensing of Hydrogen Using Templated Control of Nano-hydride Geometry and Composition

Hoang Mai Luong*[1], Minh Thien Pham[1], Tyler Guin[2], Richa Pokharel Madhogaria[3], Manh-Huong Phan[3], George Keefe Larsen*[2], and Tho Duc Nguyen*[1]

[1]*Department of Physics and Astronomy, University of Georgia, Athens, Georgia 30602, USA.*

[2]*National Security Directorate, Savannah River National Laboratory, Aiken, South Carolina 29808, USA.*

[3]*Department of Physics, University of South Florida, Tampa, Florida 33620, USA.*

*E-mail: hoanglm@uga.edu, george.larsen@srnl.doe.gov, ngtho@uga.edu

Table of Contents

S1. The thickness distribution simulation of nano-patchy (NP) samples 3

S1.1. Methods 3

S1.2. The calculation of volume-to-surface ratio of nano-patchy (NP) samples with different vapor incident angles (θ)... 6

S1.3. The calculation of volume-to-surface ratio of nano-patchy (NP) samples with different deposited thicknesses (θ = 50° is fixed) 8

S2. Additional structural characterization... 9

S2.1. Scanning electron microscopy (SEM) micrographs of NP samples................................ 9

S2.2. Morphological transition of Pd, PdAg, PdAu, and PdCo NPs with different deposited thickness...11

S2.3. Size dependence of $\ln(P_{\mathrm{Abs}}/P_{\mathrm{Des}})$, P_{Abs}, and P_{Des} in $\mathrm{NP}^{50}_{t_{\mathrm{Pd}}}$ films 15

S2.4. Power law fitting of response time in the $\mathrm{NP}^{50}_{t_{\mathrm{Pd}}}$ films... 21

S3. Optical properties of control samples 22

S4. Plateau pressures extraction... 24

S5. Sensor accuracy calculations 25

S6. The calculation of void coverage.. 25

S7. Phase transition behaviors of NP sample with different θ.. 26

S8. Optical properties of Pd, PdAg, PdAu, and PdCo composite NP upon hydrogenation and their sensing performances.. 27

S9. Noise evaluation 34

S10. PdCo NP^{50}_{5} and PdCo NP^{50}_{5}/PMMA sensors stability... 36

S11. Sensing performances of a fresh PdCo NP^{50}_{5}/PMMA sensors.. 41

S12. Sensing metrics of state-of-art optical hydrogen sensor (at room-temperature) 42

Supplementary References.. 43

S1. The thickness distribution simulation of nano-patchy (NP) samples

S1.1. Methods

We estimate the thickness distribution, surface, and volume of the hemisphere cap, based on a simple simulation including a uniform vapor flux approaches in direction $\hat{l}(\theta_0, \varphi_0)$ to an array of hexagonal close-packed nanosphere with diameter D = 500 nm (Supplementary Fig. 1a).[1,2] The thickness distribution of the nanosphere O (highlighted in orange) is calculated by considering the shadowing effects from 36 nearest-neighbor nanospheres. The surface of nanosphere O is broken down into smaller surface elements, and each of them is labelled by the polar coordinate of its center (θ, φ), where $\theta = i\Delta\theta$, $\varphi = j\Delta\varphi$, $\Delta\theta = \Delta\varphi = 0.5°$, i = 0, 1, …, 360, j = 0, 1, …, 720.

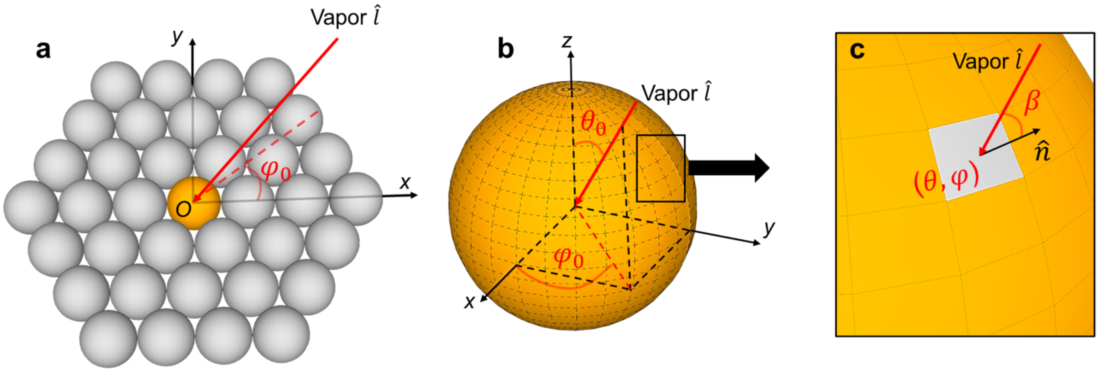

Supplementary Figure 1. (a) A cartoon illustrates the vapor deposition on an array of nanospheres. (b) The surface of nanosphere O split into several surface elements. (c) A surface element on the nanosphere O.

Our simulation is based on these following assumptions:

- Only the shadowing effect and material accumulation are considered. Other physical processes, such as surface diffusion or material penetration, are neglected.
- The deposition at different surface elements happens simultaneously as long as they are directly exposed to vapor.

- The as-deposited film is non-porous and is uniform within each surface element.

In our experimental metal deposition, the substrate holder was rotated azimuthally at a constant rate of 30 rpm to thoroughly cover the top surface of nanosphere. Therefore, in order to mimic this process, we break down the simulation into 3600 steps. We start at φ_0 = 0° (step index k_i = 1), a 0.1° azimuthal rotation of $\hat{l}(\theta_0, \varphi_0)$ happens at the end of each step, until a round of azimuthal rotation is completed (φ_0 = 359.9°) (step index k_f = 3600). In each simulation step, the thickness at each surface element $h(\theta, \varphi)$ is updated,

$$h_{k+1}(\theta, \varphi) = h_k(\theta, \varphi) + \Delta h_k. \tag{1}$$

The change of thickness for each step Δh_k is determined by whether surface element (θ, φ) is directly exposed to the vapor flux or not:

- If the surface element (θ, φ) is under the shadow of other structures (deposited materials on neighboring bead in the previous steps are also considered), then $\Delta h_k = 0$.
- If the surface element (θ, φ) can receive vapor, then $\Delta h_k = \frac{\Delta m}{\rho \cdot S(\theta,\varphi)}$, where Δm is the mass of material deposited on surface element (θ, φ) within time Δt, ρ is the density of the material, and $S(\theta, \varphi)$ is the area of surface element (θ, φ).

The vapor flux Φ is defined as,

$$\Phi = \frac{\Delta m}{\Delta t \cdot S_N} = const, \tag{2}$$

where S_N is the projection of $S(\theta, \varphi)$ onto the plane perpendicular to the vapor flux $\hat{l}(\theta_0, \varphi_0)$. With β is the angle between $\hat{l}$ and the surface normal vector $\hat{n}$ (Supplementary Fig. 1c), S_N can be written as,

$$S_N = S(\theta, \varphi)\cos\beta. \quad (3)$$

Combining Supplementary Equations (1)–(3), we achieve,

$$\Delta h_k = \frac{\Phi}{\rho}\Delta t \cos\beta. \quad (4)$$

For the simulation of $\mathrm{NP}_{t_{\mathrm{Pd}}}^{\theta_0}$ sample, in each step k, we set $\frac{\Phi}{\rho}\Delta t = \frac{t_{\mathrm{Pd}}}{k_f} = \frac{t_{\mathrm{Pd}}}{3600}$ (nm). After the last simulation step of k_f, a 3-dimension hemisphere cap are rendered based on the thickness distribution on the nanosphere O, and the volume and surface area of the patchy particle are simply obtained by using double and triple integral built-in function of MATLAB. The results are presented in Supplementary Figs. 2 and 3.

S1.2. The calculation of volume-to-surface ratio of nano-patchy (NP) samples with different vapor incident angles (θ)

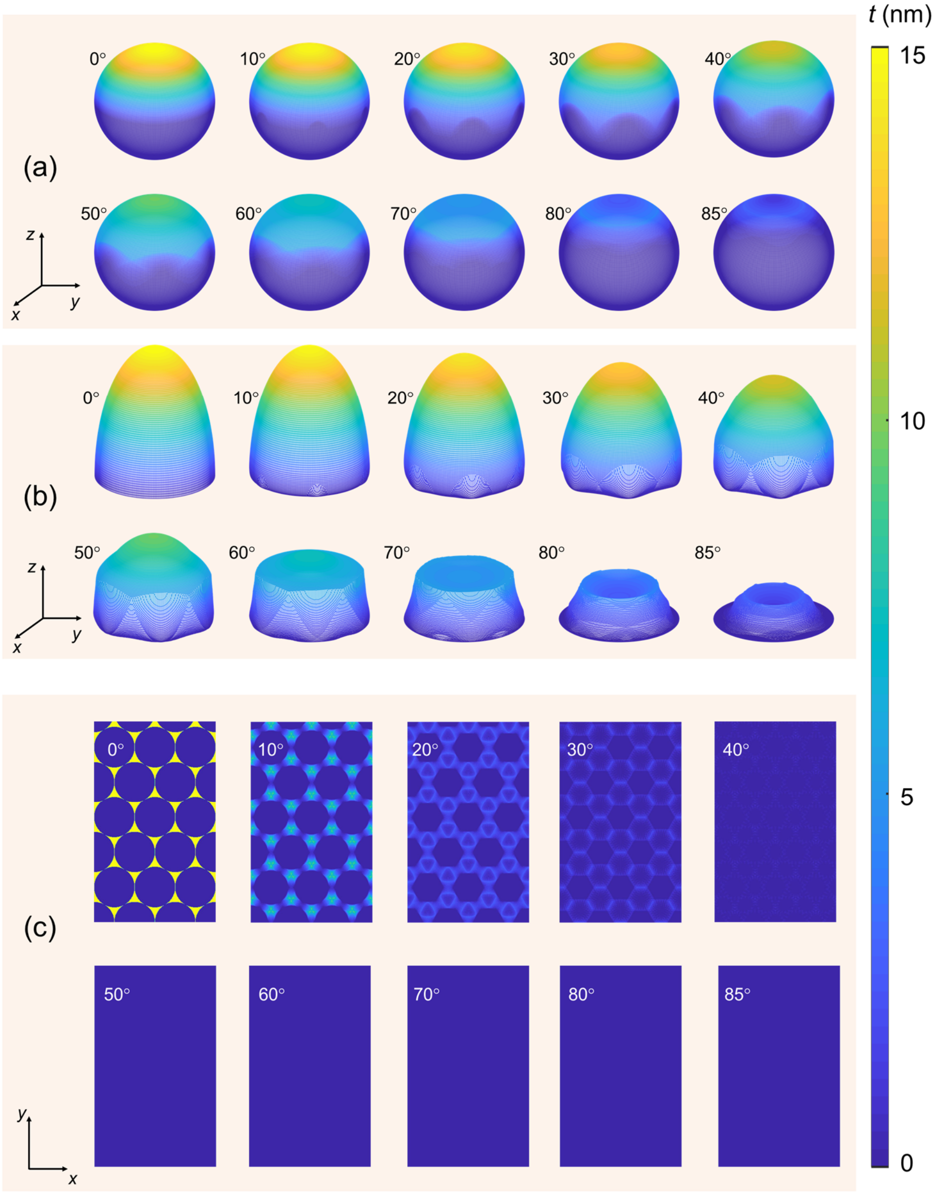

Supplementary Figure 2. (a) Simulated NP morphologies with (b) corresponding thickness distribution on polystyrene (PS) nanosphere projected to a flat surface, and (c) corresponding nano-patterns formed on glass substrate, with different vapor incident angles, θ (the deposited thickness is fixed at t = 15 nm). Note that in (b) z-axis is not-to-scale with x-axis and y-axis.

Supplementary Figure 2 presents the simulated morphology of NP under different vapor incident angles, θ (at t = 15 nm), which shows a decrease maximum deposited thickness on PS nanosphere when θ increases. We further estimate the surface area and the volume of NP with respect to the vapor incident angle as shown in Supplementary Fig. 3a. While the volume of deposited material gradually decreases when θ increases, we observe a sharp drop of surface area when θ ≥ 70°. Volume-to-surface ratio (VSR, orange curve in Supplementary Fig. 3b) is consequently calculated using these values in Supplementary Fig. 3a. We also estimate VSR of the NP in the case it is projected into a flat surface, which show about two times large of VSR in comparison with the one on PS nanosphere (pink curve in Supplementary Fig. 3b), regardless of θ.

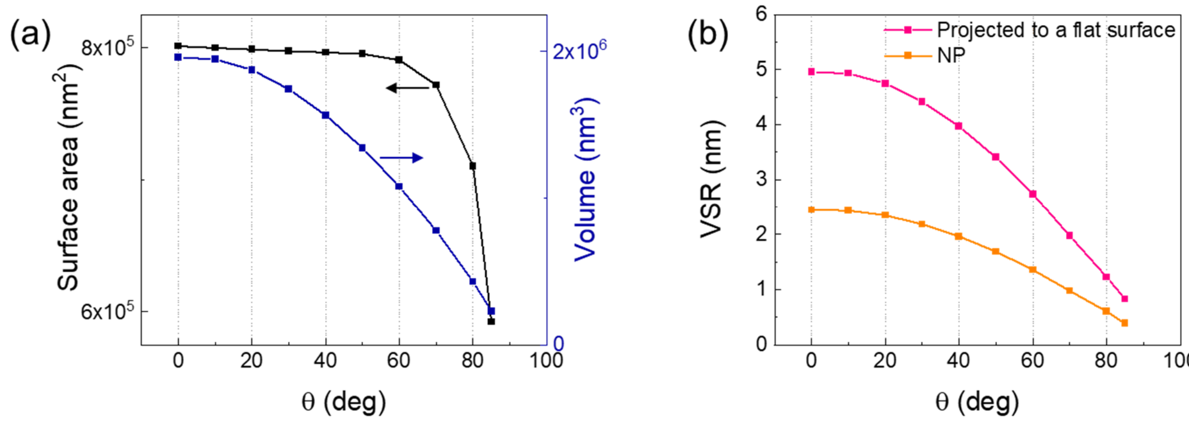

Supplementary Figure 3. (a) Estimated surface area and volume of NP sample with respect to the vapor incident angle θ and (b) estimated VSR of thickness distributions in Supplementary Figs. 2a and 2b.

S1.3. The calculation of volume-to-surface ratio of nano-patchy (NP) samples with different deposited thicknesses (θ = 50° is fixed)

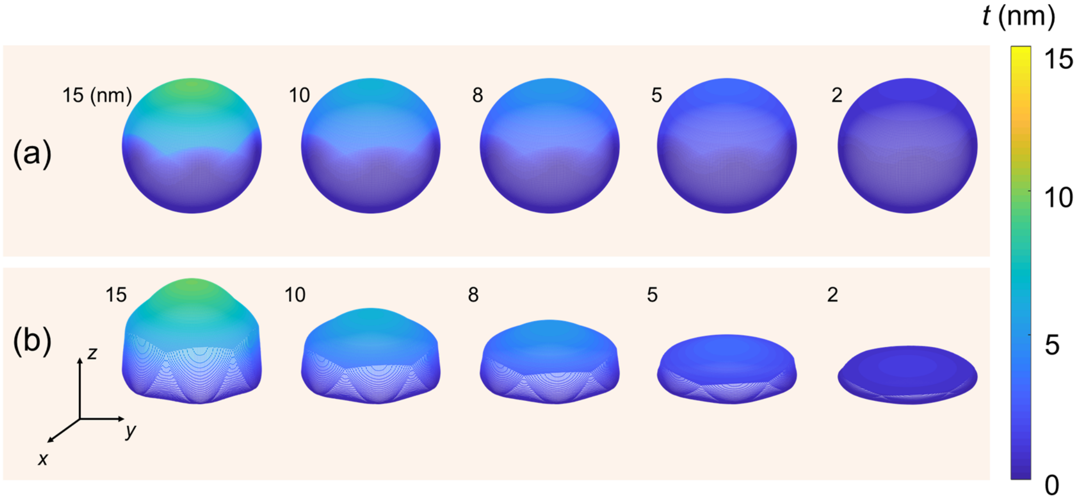

Supplementary Figure 4. (a) Simulated NP morphologies with different deposited thickness *t* (the vapor incident angle is fixed at θ = 50°) and (b) corresponding deposited thickness distribution on polystyrene (PS) nanosphere projected to a flat surface. Note that in (b) z-axis is not-to-scale with *x*-axis and *y*-axis.

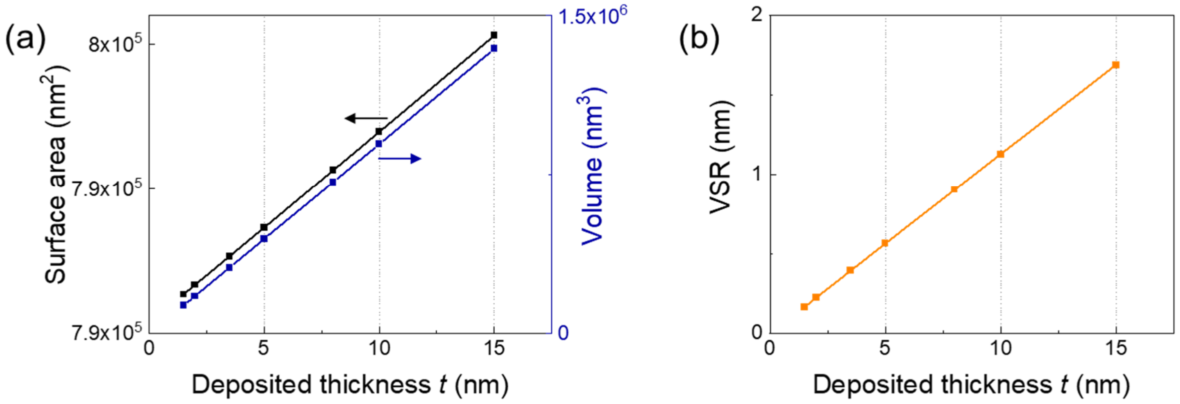

Supplementary Figure 5. (a) Estimated surface area and volume of NP sample with respect to the deposited thickness *t* and (b) estimated VSR of thickness distributions in Supplementary Fig. 4a.

S2. Additional structural characterization

S2.1. Scanning electron microscopy (SEM) micrographs of NP samples

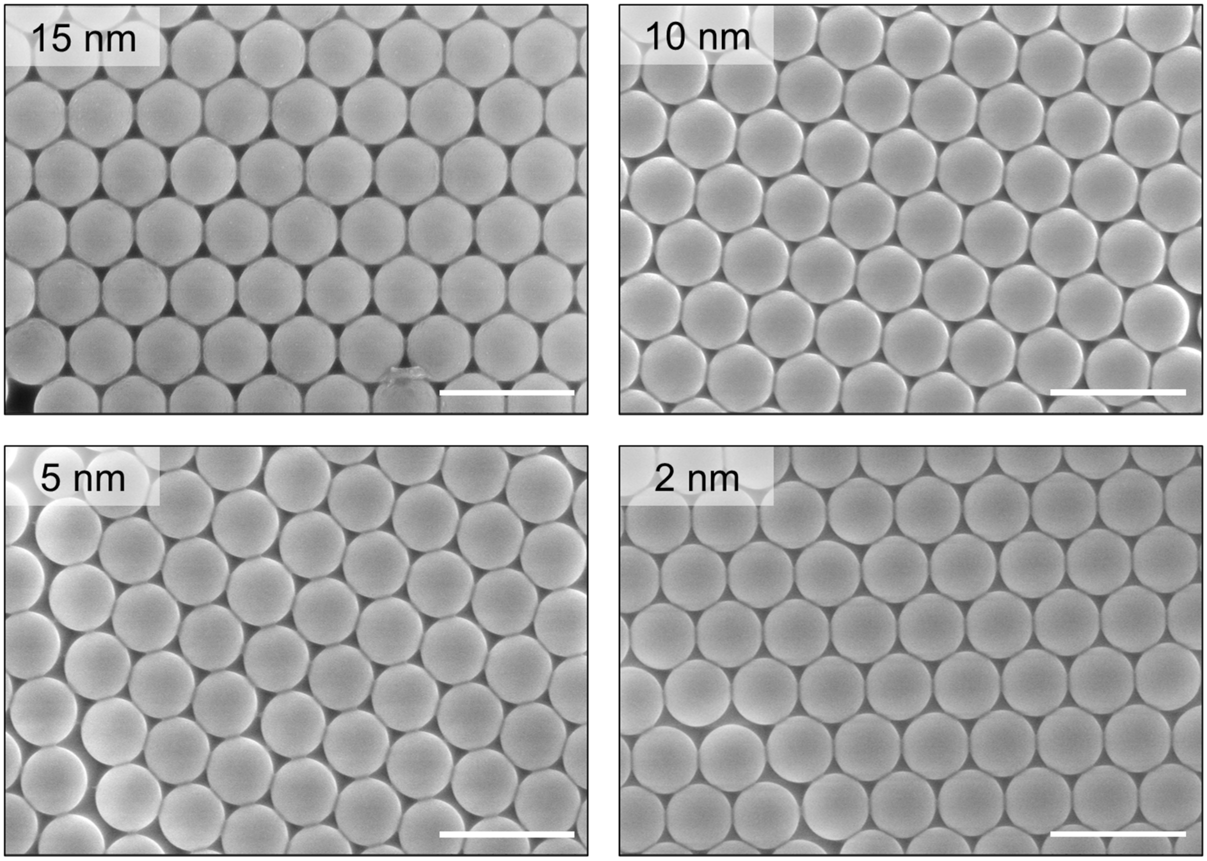

Supplementary Figure 6. Top-view scanning electron microscope (SEM) images of Pd NP_t^{50} sample with different deposited thicknesses (t = 2, 5, 10 and 15 nm). Scale bars, 1000 nm.

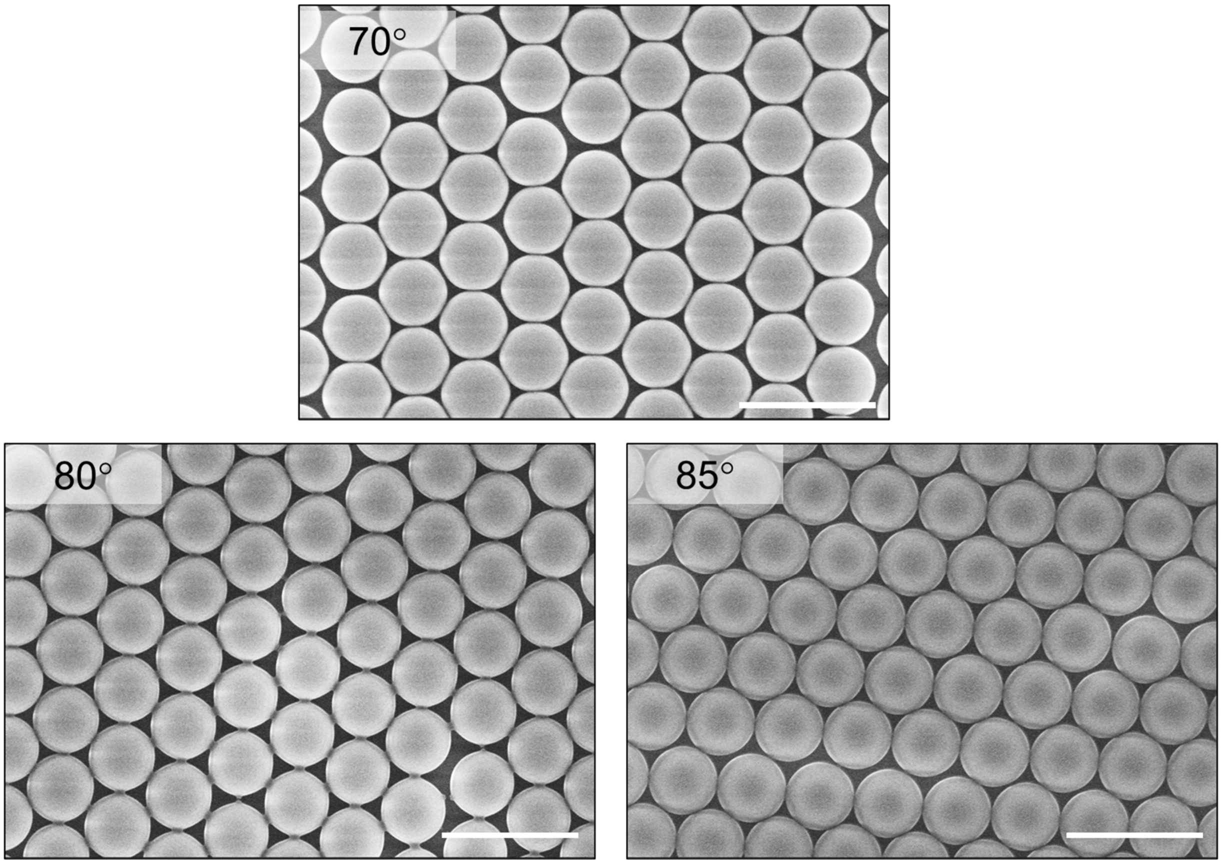

Supplementary Figure 7. Top-view scanning electron microscope (SEM) images of Pd NP_{15}^{θ} samples with different θ = 70°, 80°, and 85°. Scale bars, 1000 nm.

S2.2. Morphological transition of Pd, PdAg, PdAu, and PdCo NPs with different deposited thickness

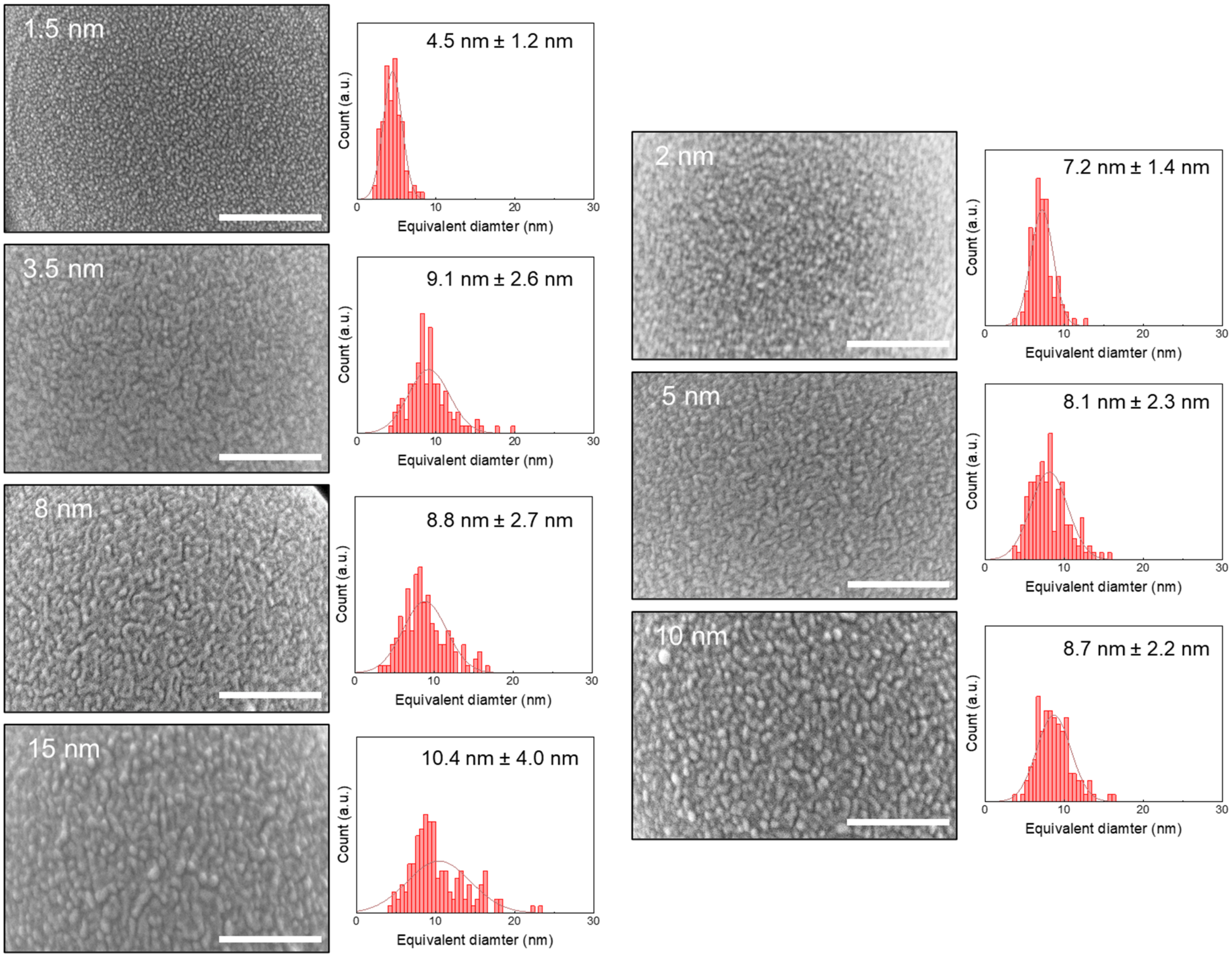

Supplementary Figure 8. Ultra-high-resolution SEM images and associated grain size analysis, showing morphology differences of Pd NP with different deposited thicknesses (t_{Pd} ranges from 1.5 to 15 nm). Scale bar: 100 nm.

The morphological transition of Pd $NP^{\theta}_{t_{Pd}}$ with increasing t_{Pd} was observed using ultra-high-resolution SEM (SU-9000, Hitachi), as revealed in Supplementary Fig. 8. The morphology of $NP^{50}_{1.5}$ contains many sub-10-nm granules, and these granules cover fully the top surface of the polystyrene nanosphere. The size of the granules grows when t_{Pd} = 2 nm, and the coalescence via

a neck (or bridge) connection between neighboring clusters can be noticed at the thickness of t_{Pd} = 3.5 nm. A continuous film is formed at a thickness of 5 nm, as these bridge connections are successively grown (a cross-sectional SEM image and EDS elemental maps of NP_5^{50} can be found in Figure 1d). Once the continuous film is shaped at a thickness of 5-nm, another distinct coalescent mechanism is observed: the size of the clusters increases as the thickness increases, associated with the reduction of the cluster density.

In order to quantify these effects, we define a volume fraction, V_f, such that

$$V_f = V_{dep}/V_{meas} \quad (5)$$

$$V_{dep} = \frac{4\pi}{6}\left(\left(R + t_{dep}\right)^3 - R^3\right) \quad (6)$$

$$V_{meas} = \frac{4\pi}{6}((R + t_{meas})^3 - R^3), \quad (7)$$

where V_{dep} is the hemispherical volume of material deposited based on a deposition of material thickness, t_{dep}, (as determined by the quartz crystal microbalance during the fabrication) onto a sphere of radius, R (R = 500 nm in this case). V_{meas} would be the volume of a hemispherical shell on a sphere of radius R if the thickness of the shell, t_{meas}, matched the average grain size measured in the high-resolution SEM images (Supplementary Fig. 8 above). Supplementary Figure 9a shows the calculated V_f for the different Pd films, and while the standard deviation, σ, is rather large for the calculations, a general trend can be observed. Consistent with the qualitative description above, films with $t_{\mathrm{Pd}} \geq 3.5$ nm have a volume fraction that exceeds the percolation threshold p_c for bond percolation ($p_c = 0.347296$), and films with $t_{\mathrm{Pd}} \geq 5$ nm have a volume fraction that exceeds p_c for site percolation ($p_c = 0.5$).[3] In this case, we consider a film to be island-like if $V_f < 0.5$ and film-like if $V_f \geq 0.5$.

As shown in Supplementary Fig. 10, different alloying elements (Ag, Au, Co) impact the nanoparticle morphology differently. Therefore, it is also interesting to compare the effects of the different alloying elements on V_f. Supplementary Figure 9b presents the V_f for the different Pd alloy films. The general trend for V_f is PdAg < Pd < PdAu < PdCo, with Pd ≈ PdAg and PdAu ≈ PdCo more loosely. It is worth noting that these films all have $V_f \geq 0.5$, but also have large σ values, where Pd and PdAg have the largest variations.

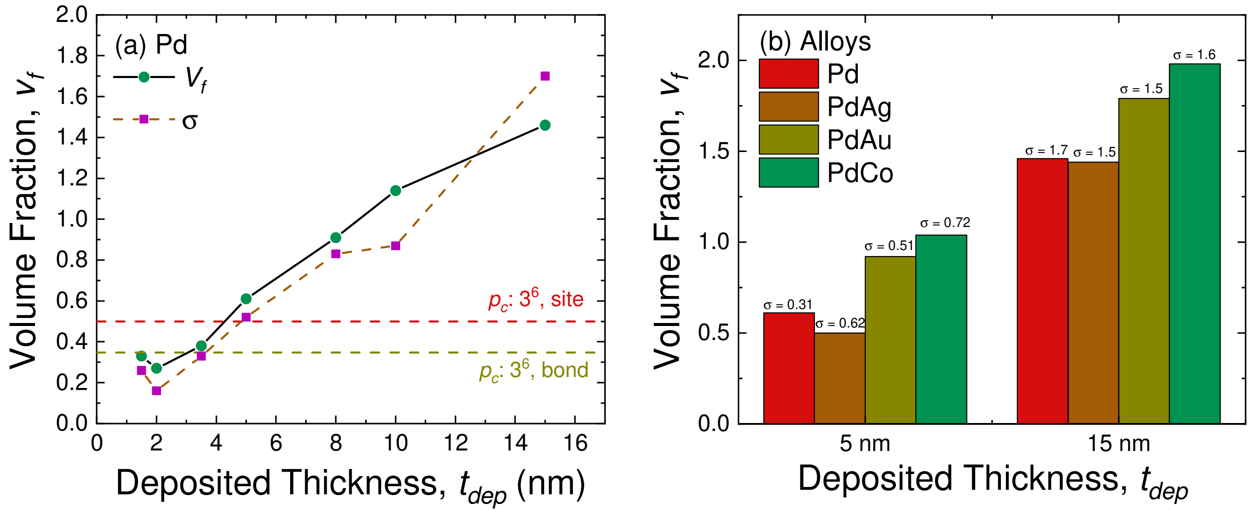

Supplementary Figure 9. Calculated volume fraction, V_f, of (a) the Pd samples versus deposited thickness and (b) the different Pd alloys for t_{dep} = 5 and 15 nm. The values of standard deviation, σ, for the V_f calculations are illustrated by the pink squares and placed above the bars in (a) and (b), respectively.

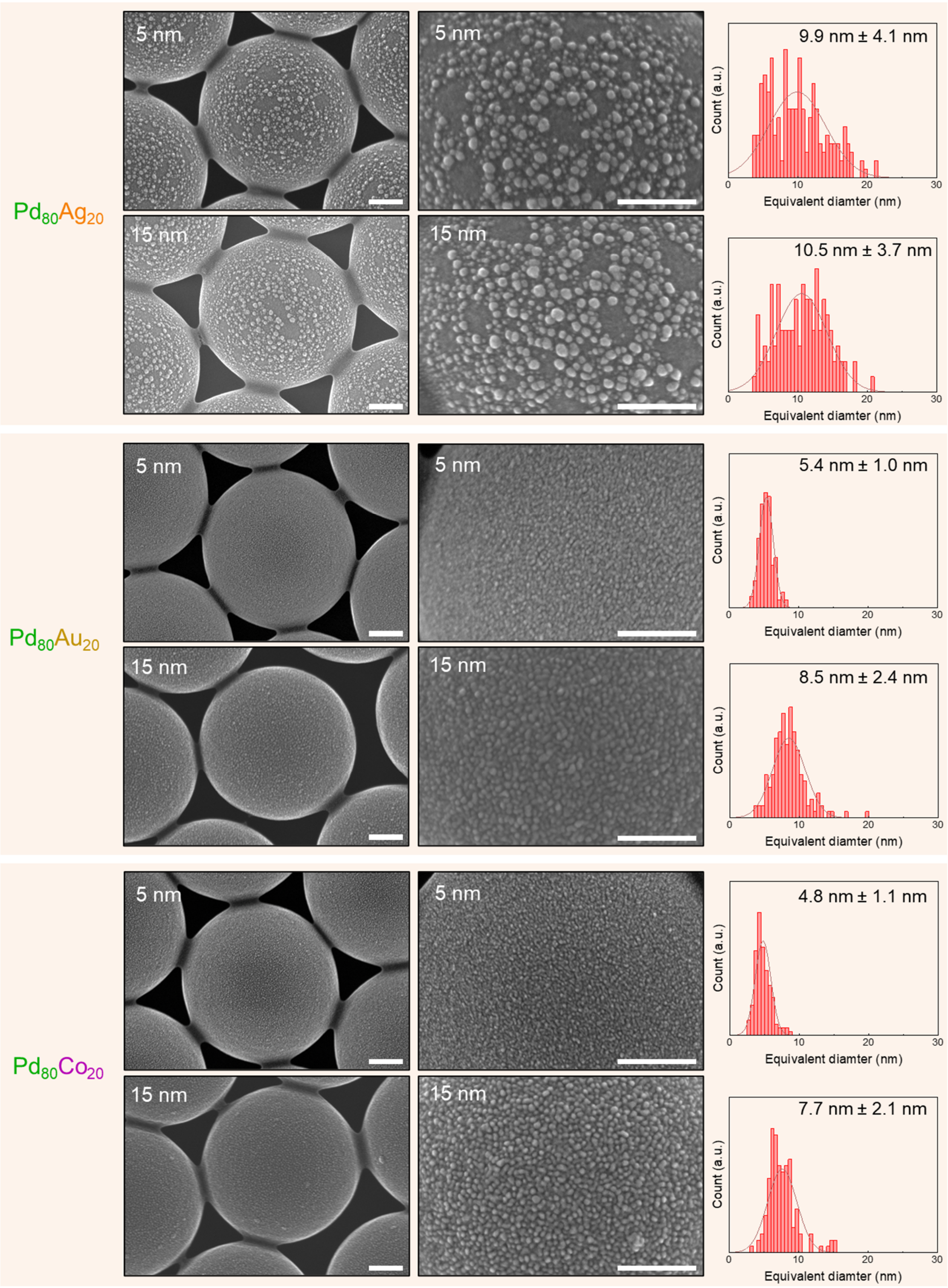

Supplementary Figure 10. Ultra-high-resolution SEM images and associated grain size analysis, showing morphology differences of NP with different deposited thicknesses (5 and 15 nm) and compositions. Scale bar: 100 nm.

S2.3. Size dependence of ln(P_{Abs}/P_{Des}), P_{Abs}, and P_{Des} in $NP_{t_{Pd}}^{50}$ films

As shown in Figure 2d in the main text, ln(P_{Abs}/P_{Des}), P_{Abs}, and P_{Des} display a size dependent behavior, where a noticeable transition occurs for t_{Pd} < 5 nm. This transition in behavior matches the transition between island-like to film-like morphologies described in Supplementary Subsection 2.2 above. Further, the size-dependent effects can be described by a size-dependent critical temperature, T_c, and an increasing effect of subsurface sites for smaller particles.

The size dependence of hysteresis in nanoparticles, which is quantitatively expressed as ln(P_{Abs}/P_{Des}), has been comprehensively analyzed by Griessen et al. and a robust scaling law has been proposed.[4] Based on a simple lattice gas model, the hysteresis was found to only depend on the ratio of T/T_c, where T_c is the critical temperature at which the hysteresis vanishes. The full-spinodal line for hysteresis is given by:

$$ln\left(\frac{P_{us}}{P_{ls}}\right) = 8\frac{T_c}{T}z + 4\,ln\left(\frac{1-z}{1+z}\right), \quad (8)$$

where $z = \sqrt{1-(T/T_c)}$ and P_{us} and P_{ls} are upper and lower spinodal pressures, respectively. Griessen et al. found that the hysteresis behavior of a wide variety of Pd nanoparticles fell between the full spinodal hysteresis line given by Supplementary Equation (8) and 45% of the full spinodal hysteresis value. The size dependence of T_c was found to be well represented by:

$$T_c = A - (B / L), \quad (9)$$

where A and B are fitting parameters and L is the size of a nanocube in nanometers. Using $T = 300$ K and Supplementary Equation (8) we can similarly extract T_c values as a function of t_{Pd} for the $NP_{t_{Pd}}^{50}$ films and determine the scaling relationship with t_{Pd}. The results assuming a full spinodal hysteresis are presented in Supplementary Fig. 11. T_c varies from 324 - 382 K with increasing size

and is well fit by Supplementary Equation (9) with A = 387.054 and B = 90.1308. While not presented here, the results for 45% spinodal hysteresis (0.45 is added as a multiplicative factor to the right-hand side (RHS) of Supplementary Equation (9)) show an increase T_c to 341 to 445 K, and are fit by Supplementary Equation (9) with A = 453.02 and B = 159.995. These values agree well with the results of Griessen et al. and provide further evidence of the universality of the relationship derived therein, as the fitting here encompasses the transition between island-like and film-like morphology. Thus, the shrinking hysteresis observed in the $\mathrm{NP}_{t_{\mathrm{Pd}}}^{50}$ films is due to a decreasing T_c value with decreasing t_{Pd}.

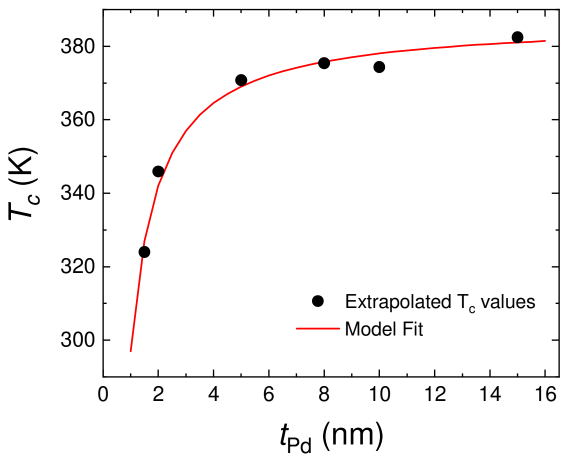

Supplementary Figure 11. T_c values as a function of Pd thickness. The data points were obtained from experimental values using Supplementary Equation (8). The model curve was generated using Supplementary Equation (9) with fitting parameters.

The $\ln(P_{\mathrm{Abs}}/P_{\mathrm{Des}})$ values can also be used to understand the effects of subsurface sites on P_{Abs}, and P_{Des}. According to Sachs et al. the fraction c_t of subsurface sites can be determined given the well-supported assumption that hydrogen in subsurface sites does not transform to a hydride phase:

$$c_t = 1 - \frac{c_\beta^{nano} - c_\alpha^{nano}}{c_\beta^{bulk} - c_\alpha^{bulk}}, \qquad (10)$$

where the superscripts denote the nanoparticle or bulk concentrations and the subscripts denote the concentrations at the boundaries of the α and β phases.[5] As described in the main text, the hysteresis can be explained by the thermodynamics of an open, coherent two-phase system. According to Schwarz and Khachaturyan, the absorption and desorption plateau pressures may then be calculated by:

$$ln\left(\frac{P_{Abs}}{P_{Des}}\right) = \left(\frac{4\Omega\ G_s\frac{1+\upsilon}{1-\upsilon}\varepsilon_0{}^2(c_\beta - c_\alpha)}{kT}\right), \quad (11)$$

where the volume of one hydrogen atom in Pd, Ω = 2.607 Å^3, the shear modulus of Pd, G_s = 47.7×10^9 Pa, the Poisson number ν = 0.385, the change in lattice constant, ε_0 = 0.063, and Boltzmann constant k.[6] Using c_α^{bulk}= 0.008 and c_β^{bulk}= 0.0607, Supplementary Equation (11) gives ln(P_{Abs}/P_{Des}) = 0.62, which is in excellent agreement with the experimentally measured values for $t_{Pd} \geq 5$ nm as described in the main text. By combining Supplementary Equations (10) and (11) and using the experimentally determined ln(P_{Abs}/P_{Des}) values, the fraction of subsurface sites, c_t, can be calculated for each t_{Pd} value. The values are < 0.10 for $t_{Pd} \geq 5$ nm but rapidly increase for $t_{Pd} < 5$ nm (Supplementary Fig. 12). The volumes of the bulk and subsurface regions in the film-like hemispherical patchy caps ($t_{Pd} \geq 5$ nm) may be respectively estimated as:

$$V_b = \frac{4\pi}{6}((R + t_{Pd} - h)^3 - R^3), \quad (12)$$

$$V_{ss} = \frac{4\pi}{6}((R + t_{Pd})^3 - (R + t_{Pd} - h)^3), \quad (13)$$

$$V_{tot} = V_{ss} + V_b, \quad (14)$$

where V_{tot} is the total volume, the radius of the PS bead template is R = 500 nm, and h is the thickness of the subsurface layer. Values for h in the literature range from 0.3 - 4 nm, depending

on the structure and Pd crystalline surface.[4] By setting $c_t = V_{ss} / V_{tot}$ and solving for h, we find that $h = 0.4$ nm for $t_{\mathrm{Pd}} \geq 5$ nm, which agrees very well with the literature values.

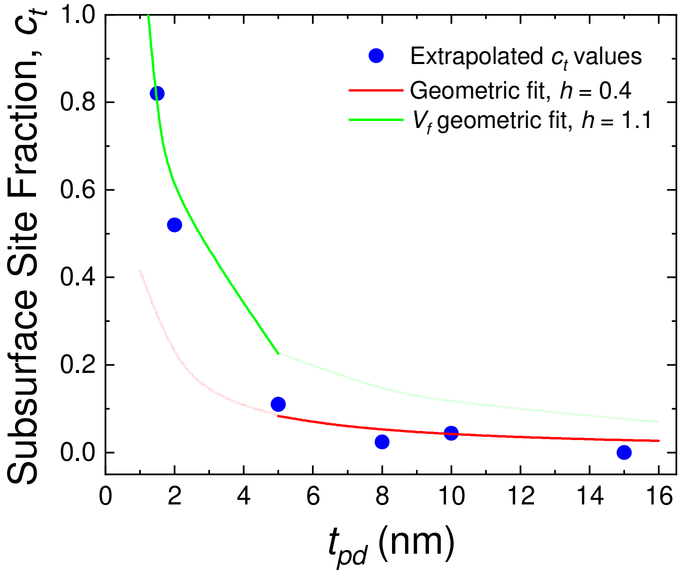

Supplementary Figure 12. Subsurface sites as a function of Pd thickness. The data points were obtained from experimental values using Supplementary Equation (11). The model curves were generated using Supplementary Equations (12)–(14) with h = 0.4 nm or by volume fraction modification with $h = 1.1$ nm.

As described in Supplementary Subsection 2.2 above, the Pd films transition from a film-like morphology to island-like morphology for $t_{\mathrm{Pd}} < 5$ nm. In order to estimate the subsurface site layer thickness, we modify our assumptions such that $c_t = (1/V_f)\ (V_{ss} / V_{tot})$ and by using t_{meas}, instead of t_{Pd} in Supplementary Equations (12)–(14), where V_f and t_{meas} are defined in Supplementary Subsection 2.2. In this case, we find h = 1.1 nm provides a good fit for $t_{\mathrm{Pd}} < 5$ nm. It is worth noting a spherical calculation of h based on t_{meas} indicates $h = 2$ nm and 1.6 nm for $t_{\mathrm{Pd}} = 1.5$ nm and 2 nm respectively.

Evaluating the experimental data through Supplementary Equations (10)–(13) enabled quantification of the increasing relative volume of subsurface sites as the Pd film thickness decreases in the patchy particles. The importance of these sites on the thermodynamics of adsorption increases as the particle size gets smaller. Wadell et al. suggested that the behavior of the enthalpy of hydride formation as Pd nanoparticles shrink is a combination of the competitive effects of surface tension and subsurface sites.[7] That is, the surface tension contribution decreases as particles shrink, while the relative number subsurface sites, which have a higher absorption energy than bulk sites, increases. To quantify these effects, they developed an analytical Langmuirian-type model, where the chemical potentials of H atoms in the bulk hydride and subsurface volumes respectively are:

$$\mu_b^H = -E_0 + kT\, ln\left(\frac{\theta_b}{1-\theta_b}\right) + \frac{2\gamma\Omega}{r}, \tag{15}$$

$$\mu_{ss}^H = -E_0 - \Delta E + kT\, ln\left(\frac{\theta_{ss}}{1-\theta_{ss}}\right) + \frac{2\gamma\Omega}{r}. \tag{16}$$

θ_b and θ_{ss} are the H coverages of the bulk and subsurface sites, E_0 is the gain in energy during the hydride formation at R $\rightarrow \infty$, $\Delta E > 0$ is the energy difference between the bulk and subsurface sites, γ is the surface tension (0.2 eV/Å^2 for Pd), and Ω is the partial volume of hydrogen in the hydride phase (Ω = 2.607 Å^3). The last terms on the RHS of Supplementary Equations (15) and (16) are the surface tension term, and for the Pd patchy system $r = R + t_{Pd}$, with R = 500 nm. At equilibrium, the chemical potential of H_2 molecules in the gas phase is:

$$\mu_{H_2} = kT\, ln\left[\left(\frac{P}{(kT^{5/2})}\right)\left(\frac{2\pi\hbar^2}{m}\right)^{3/2}\right], \tag{17}$$

$$\mu_{H_2} = 2\mu_b^H = 2\mu_{ss}^H, \tag{18}$$

where m is the mass of a H_2 molecule and $\hbar$ is the reduced Planck's constant. Note that this expression takes only the translation partition function into account, and the minor rotational and vibrational contributions are ignored. The average coverage of the bulk and subsurface sites is given by:

$$\langle\theta\rangle = \frac{V_b}{V_{tot}}\theta_b + \frac{V_{ss}}{V_{tot}}\theta_{ss} \,. \qquad (19)$$

By setting $\langle\theta\rangle = 0.5$, using the value for h determined above for ($h = 0.4$nm), selecting $\Delta E = 2.6$ kJ/mol H, and using the experimentally measured absorption plateau pressures at $T = 300$ K, Supplementary Equations (15)–(19) can be used to solve for θ_b, θ_{ss}, and E_0 for the $t_{Pd} > 5$ nm values. The fittings result in average values of $\theta_b = 0.48 \pm 0.01$, $\theta_{ss} = 0.971 \pm 0.001$, and $E_0 = 19.6 \pm 0.1$ kJ/mol H. The obtained value of $E_0 \approx 20$ kJ/mol H in excellent agreement with literature values.[4,7] On the other hand, θ_{ss} is higher than expected ($\theta_{ss} \times 0.607 = 0.44$ H/Pd, typically), and thus could indicate higher H capacity in surface shell sites facilitated by the polymer substrate. Similar effects were seen in Pd nanocubes covered with a metal organic framework.[8] It is interesting to note that the subsurface site effect is more pronounced in the Pd patchy particles than it is in many other Pd nanoparticles. This is because the surface curvature changes much more slowly than the volume to surface ratio in the Pd patches. Hence, Wadell et al. observed a relatively constant enthalpy of hydride formation for decreasing particle size in their spherical nanoparticles.

In addition, we note that the hydrogen absorption and desorption isotherm pressures above the critical thickness ($t_{Pd} \approx 5$ nm) are $P_{Abs} \approx$ 13-14 mbar and $P_{Des} \approx 7$ mbar, respectively, both of which are appreciably smaller than those of Pd thin films and Pd nano-particles on flat substrates ($P_{Abs} \approx$ 22-25 mbar and $P_{Des} \approx$ 10-11 mbar).[9,10] The lowering of plateau pressures may arise from in-plane strain imposed by the curvature of the substrate, which increases the long-range attractive H–H

interactions.[11,12] Alternatively, a reduced adhesive force between the Pd structures and the softer PS substrate may also contribute to the lowering of the plateau pressures relative to Pd structures supported on rigid substrates.[12]

S2.4. Power law fitting of response time in the $NP_{t_{Pd}}^{50}$ films

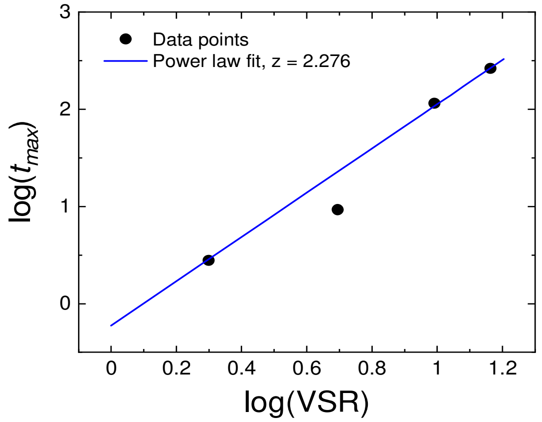

Supplementary Figure 13. Power law fitting ($t_{max} = a \times VSR^z$) of the maximum response times of the $NP_{t_{Pd}}^{50}$ films. The exponent value of $z \approx 2.3$ agrees well with the diffusion limited case in metal hydride systems.[13] Note that VSR will go as length L or radius r in nanocubes and nanoparticles, which are traditionally used in power law fittings. In the Pd hemispherical caps on 500 nm PS beads VSR will be close to t_{Pd} for small thicknesses (< 20 nm), but will begin to deviate for increasing thicknesses.

S3. Optical properties of control samples

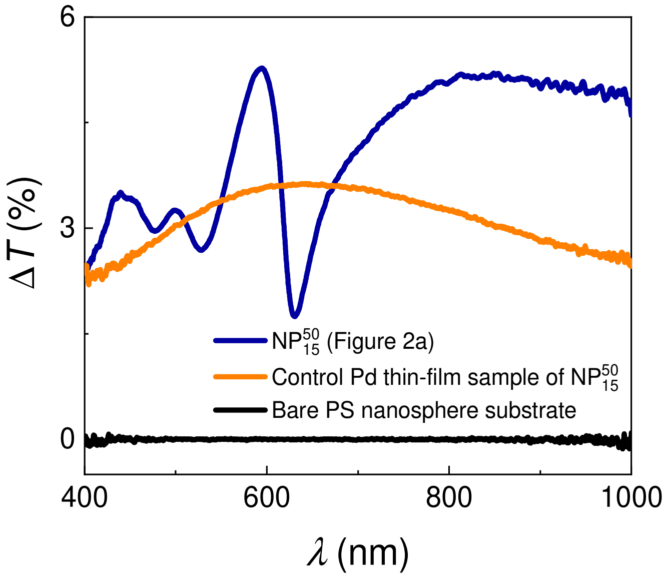

Supplementary Figure 14. Optical transmission change $\Delta T(\lambda) = T_{1000\,\mathrm{mbar}} - T_{0\,\mathrm{mbar}}$ of PS nanosphere monolayer substrate, NP$^{50}_{15}$ sample, and corresponding control Pd thin-film sample (t_{Pd} = 15 nm, θ = 50°).

The maximum optical change in the NP sample is much greater than that of the control Pd thin film sample (Supplementary Fig. 14). This can be attributed to diffraction within the hexagonal Pd patchy arrays, which does not occur in a thin film. Transmitted light at certain wavelengths is diffracted, partially localized inside the PS nanosphere causing the so-called partially-localized surface plasmon resonance (LSPR), and then is amplified through interactions with the Pd hemisphere cap.[14,15] This LSPR is suppressed when Pd turns to Pd hydrides, inducing significant redistribution of the local electric field.[14] This enhanced light-material interaction emerges in the far-field as local extrema on ΔT spectra. To confirm this hypothesis, FDTD calculations were performed using NP morphology generated by a home-built MATLAB program,[1] and the calculated results are in excellent agreement with the experimental transmission spectra

(Supplementary Figs. 15b–c). The FDTD calculated time-averaged intensity $|E/E_0|^2$ maps (square of ratio of electric fields E to the incident field E_0), extracted at the NPs cross-section at $\Delta T(\lambda)$ peaks, indicates enhanced localized electric field under the Pd patchy cap (Supplementary Figs. 15d–f), confirming the near- to far-field process.

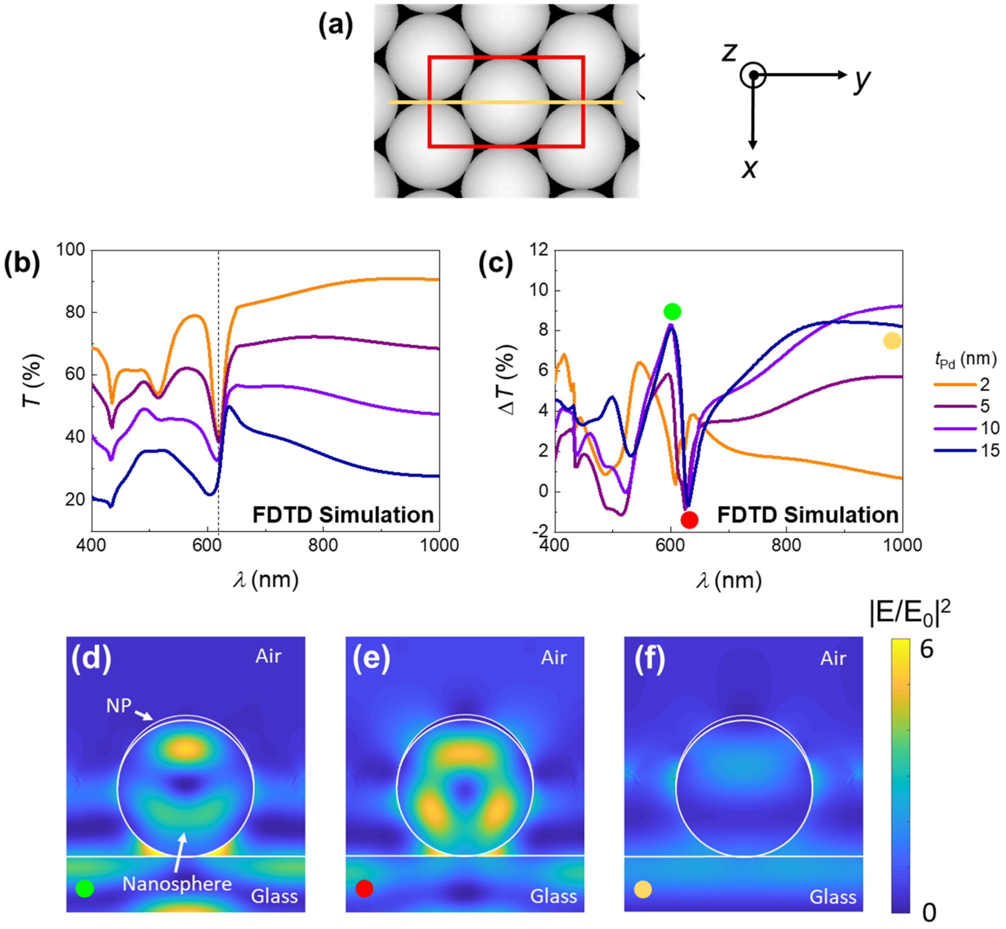

Supplementary Figure 15. (a) A top-view of the hexagonal lattice of NP, the red box denotes the rectangular unit cell for FDTD calculations. (b) FDTD calculated optical transmission spectra $T(\lambda)$ of NP sample, when P_{H_2} = 0 mbar. (c) FDTD calculated $\Delta T(\lambda) = T_{1000\,mbar}$ - $T_{0\,mbar}$ spectra of NP samples. (d-f) Time-averaged intensity maps of the FDTD calculated local electric field at the cross-section plane denoted by the yellow line (Supplementary Fig. 15a). Circle dots with different colors

(bottom left of the figures) indicate the wavelength that the map is extracted (Supplementary Fig. 15c). We observe an partial-localized enhanced electric field spot located under the Pd NP at $\Delta T(\lambda)$ peak (at green circle, Supplementary Fig. 15c), and a whispering-gallery modes inside nanosphere at $\Delta T(\lambda)$ dip (at red circle, Supplementary Fig. 15c). On the other hand, no enhanced electric field can be observed when it is out of resonance (at yellow circle, Supplementary Fig. 15c).

S4. Plateau pressures extraction

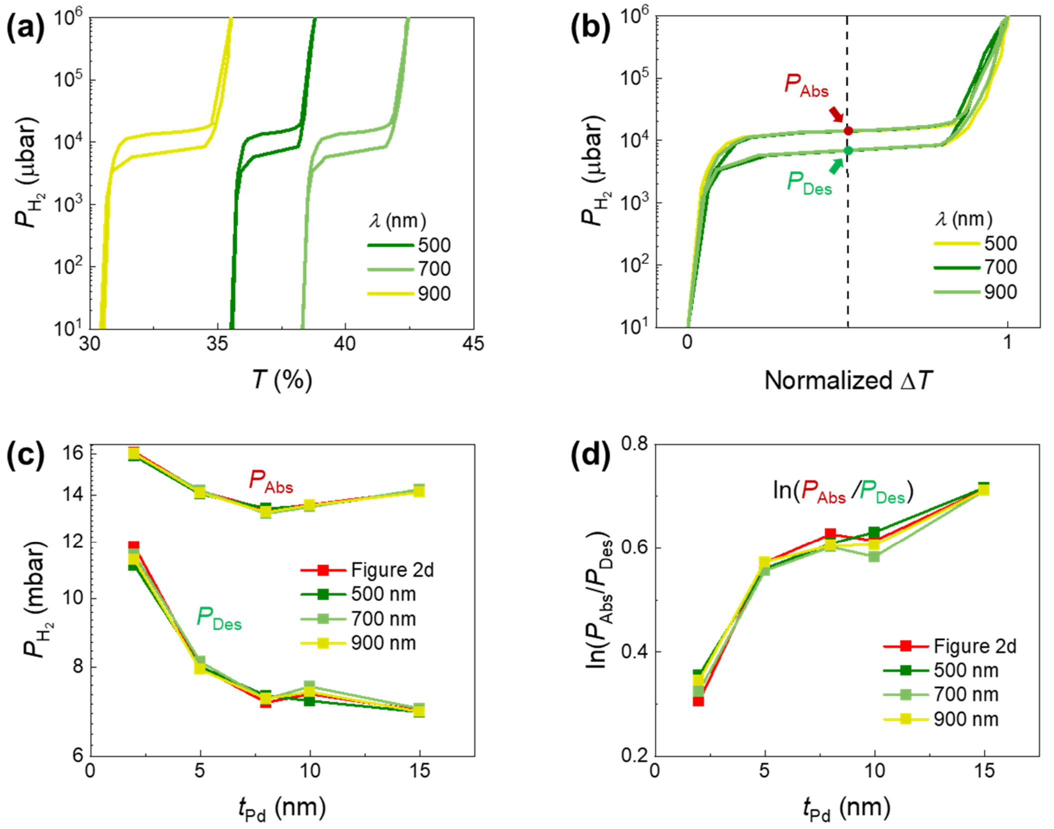

Supplementary Figure 16. (a) Optical hydrogen sorption isotherm of representative Pd NP_{15}^{50} sample, extracted at different wavelength positions and (b) normalized ΔT sorption isotherm at the corresponding wavelengths. Absorption and desorption plateau pressures (P_{Abs} and P_{Des}, respectively) are extracted at normalized ΔT = 0.5 (as denoted in Supplementary Fig. 16b). (c) P_{Abs}, P_{Des}, and (d) $\ln(P_{Abs}/P_{Des})$ extracted at different wavelength positions. We observe a slight variation of these values at different wavelengths; however, the general thickness-dependent trend is conserved.

S5. Sensor accuracy calculations

The sensor accuracy (A) is calculated by the following equation:[16]

$$A = \frac{|log(P_{\text{Abs}}) - log(P_{\text{Des}})| \times 100}{|log(P_{\text{Abs}}) + log(P_{\text{Des}})|/2}, \quad (20)$$

where P_{Abs} and P_{Des} are the pressures reading (in μbar) during hydrogen absorption and desorption, respectively.

S6. The calculation of void coverage

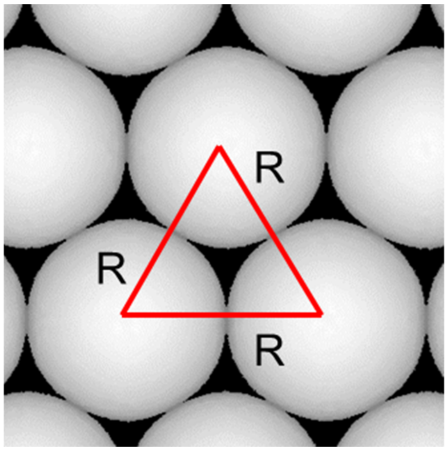

Supplementary Figure 17. The calculation configuration for void coverage.

The void coverage percentage V (black area) between PS nanosphere (radius of R) can be calculated as:

$$V = (\tfrac{1}{2} \times 2R \times \tfrac{\sqrt{3}}{2} R - 3 \times \tfrac{1}{6} \times \pi R^2)/\tfrac{1}{2} \times 2R \times \tfrac{\sqrt{3}}{2} R = \frac{\sqrt{3} - \pi/2}{\sqrt{3}} \approx 0.093$$

S7. Phase transition behaviors of NP sample with different θ

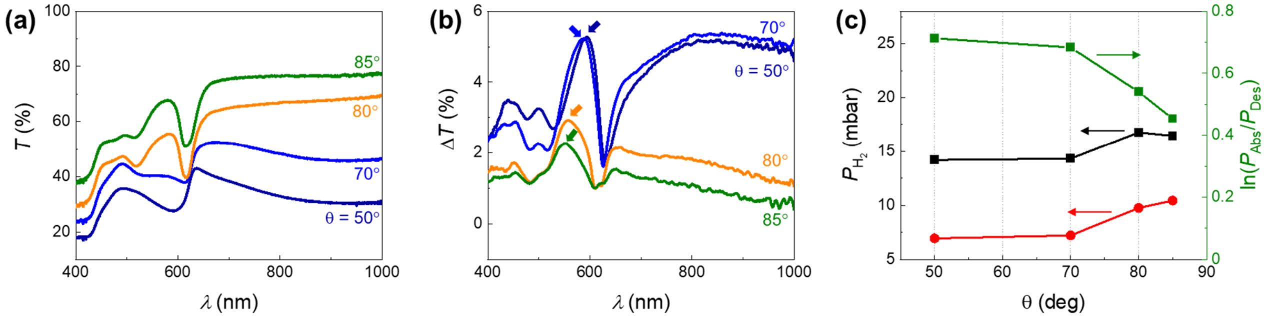

Supplementary Figure 18. (a) Experimental optical transmission spectra $T(\lambda)$ (at P_{H_2} = 0 mbar) and (b) optical transmission change $\Delta T(\lambda) = T_{1000\ mbar}$ - $T_{0\ mbar}$ of NP with different vapor incident angles of θ. (c) Extracted plateau pressures for hydrogen absorption (P_{Abs}), desorption (P_{Des}), and ln(P_{Abs}/P_{Des}) for different θ.

S8. Optical properties of Pd, PdAg, PdAu, and PdCo composite NP upon hydrogenation and their sensing performances

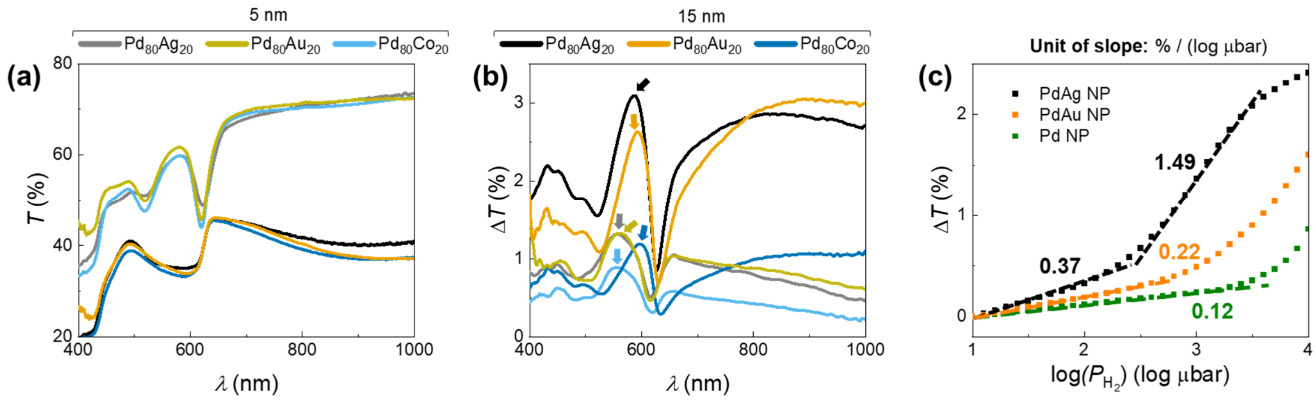

Supplementary Figure 19. (a) Experimental optical transmission spectra $T(\lambda)$ and (b) optical transmission changes $\Delta T(\lambda) = T_{1000\,mbar} - T_{0\,mbar}$ of $Pd_{80}Ag_{20}$/$Pd_{80}Au_{20}$/$Pd_{80}Co_{20}$ NP_{15}^{50} and NP_{5}^{50} samples. Colored arrows denote the position of $\Delta T(\lambda)$ spectra peaks, where sorption isotherms are extracted (results presented in Figure 4b). (c) Extracted ΔT absorption isotherm of $Pd_{80}Ag_{20}$, $Pd_{80}Au_{20}$ and Pd NP_{15}^{50} samples at spectra peak. The sensitivity numbers indicate the change in relative transmission magnitude (%) per 1 (log µbar) increase in $\log(P_{H_2})$.

In general, incorporating Ag or Au into NPs does not change the overall shape of $\Delta T(\lambda)$ (at P_{H_2} = 1000 mbar), but it reduces the absolute value of $\Delta T(\lambda)$ ~50 % in comparison to that of pure Pd NP with the same deposited thickness (Supplementary Fig. 19). The drop of $\Delta T(\lambda)$ magnitude is more significant than the amount of Pd atoms replaced by Ag or Au (20%), which can be explained by the reduction in the limiting solubility of H in the PdAg or PdAu system. This reduction is due to a decreasing number of available electron states in the d-band of the Pd electronic structure, which is induced by the introduction of Ag/Au atoms.[17] In contrast, at very low pressures (P_{H_2} < 1 mbar), the $Pd_{80}Ag_{20}$ and $Pd_{80}Au_{20}$ alloy NP sensor shows about 3- and 1.8-times enhancement in sensitivity in comparison to that of pure Pd NP (Supplementary Fig. 19c), respectively, which

implies an increase of H solubility at low pressures arising from the interaction between an interstitial hydrogen and Ag/Au in the lattice.[17]

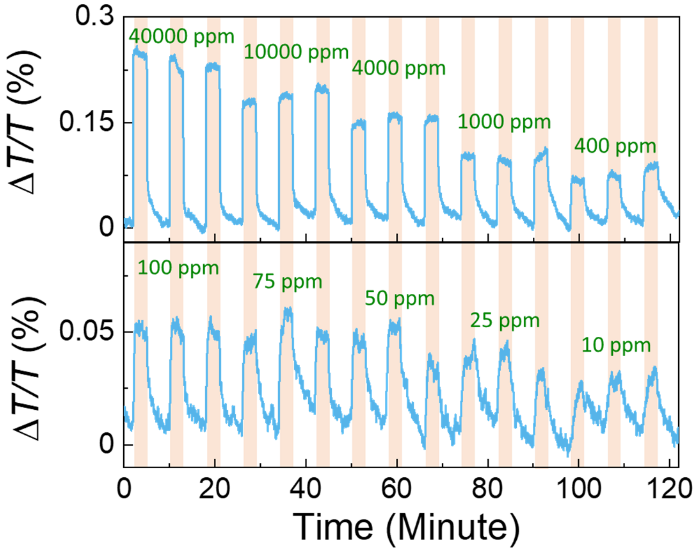

Supplementary Figure 20. $\Delta T/T$ response of $Pd_{80}Co_{20}$ (t = 5 nm) composite NPs (1.25 Hz of sampling frequency) with different hydrogen concentration (C_{H_2}), measured in flowing nitrogen (400 ml/min). Shaded areas denote the periods where the sensor is exposed to hydrogen.

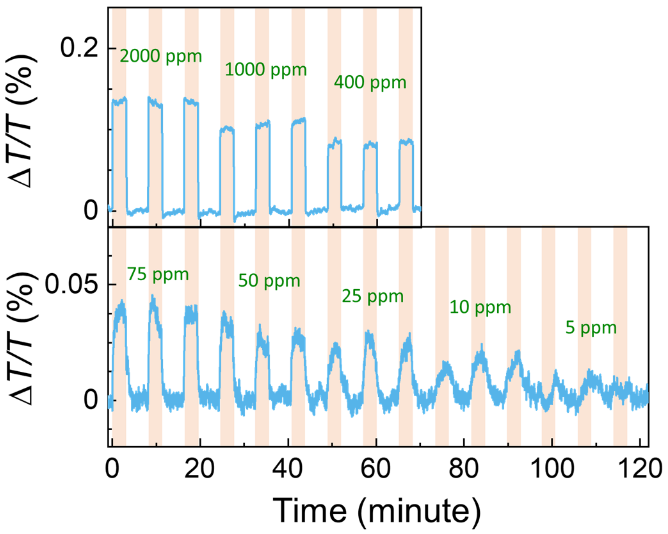

Supplementary Figure 21. $\Delta T/T$ response of $Pd_{80}Co_{20}$ (t = 5 nm) composite NPs (1.25 Hz of sampling frequency) with different hydrogen concentration (C_{H_2}), measured in flowing synthetic gas (400 ml/min). Shaded areas denote the periods where the sensor is exposed to hydrogen.

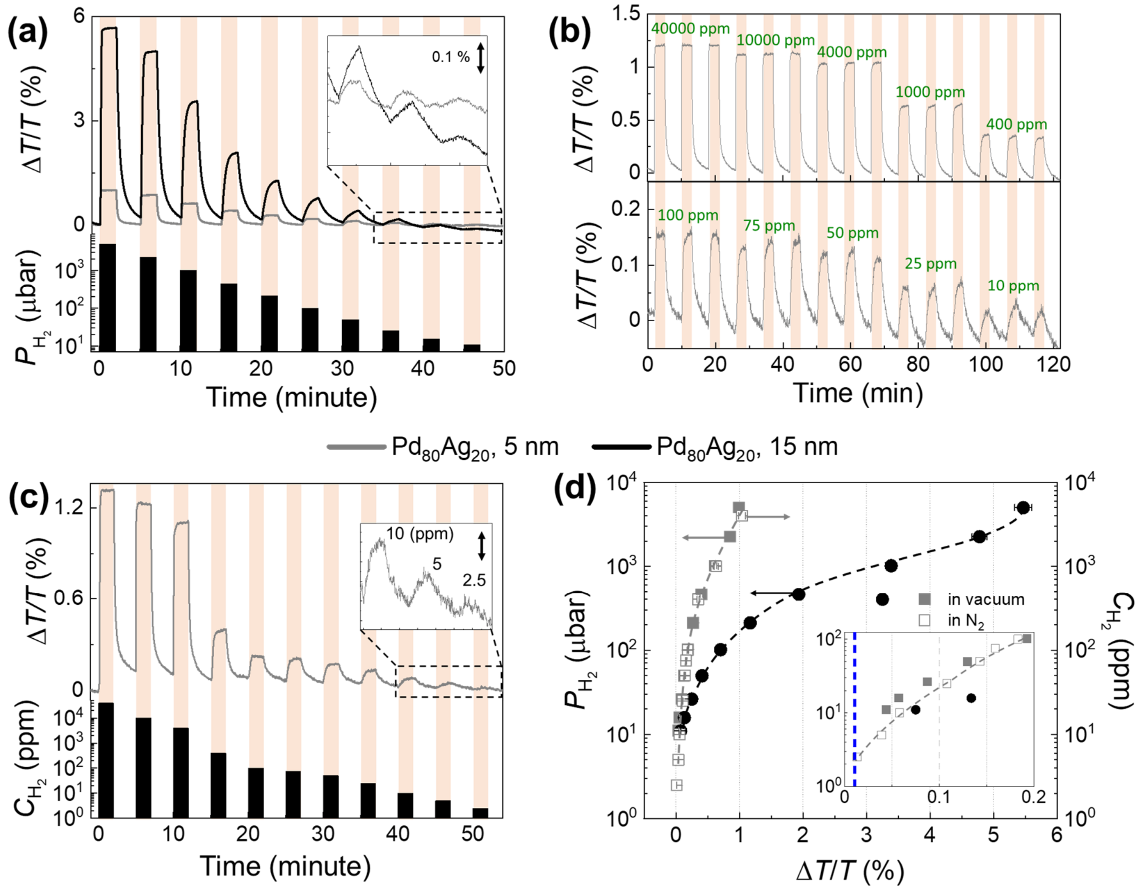

Supplementary Figure 22. (a) $\Delta T/T$ response of Pd$_{80}$Ag$_{20}$ NP^{50}_{15} and NP^{50}_{5} sensors to stepwise decreasing hydrogen pressure in the 5000 – 11 μbar range, measured at 1.25 Hz sampling frequency in a vacuum chamber. (b)-(c) $\Delta T/T$ response of Pd$_{80}$Ag$_{20}$ NP^{50}_{5} sensors with different hydrogen concentrations (C_{H_2}) of 4 % - 2.5 ppm, measured in flowing nitrogen (400 ml/min). Up-down arrows in the insets of (c) correspond to 0.03 %. Shaded areas denote the periods where the sensor is exposed to hydrogen. (d) Measured $\Delta T/T$ response as a function of hydrogen pressure/concentration derived from (a-c). The blue dashed line denotes the defined LOD at 3σ ≈ 0.013 % (see Supplementary Section 9).

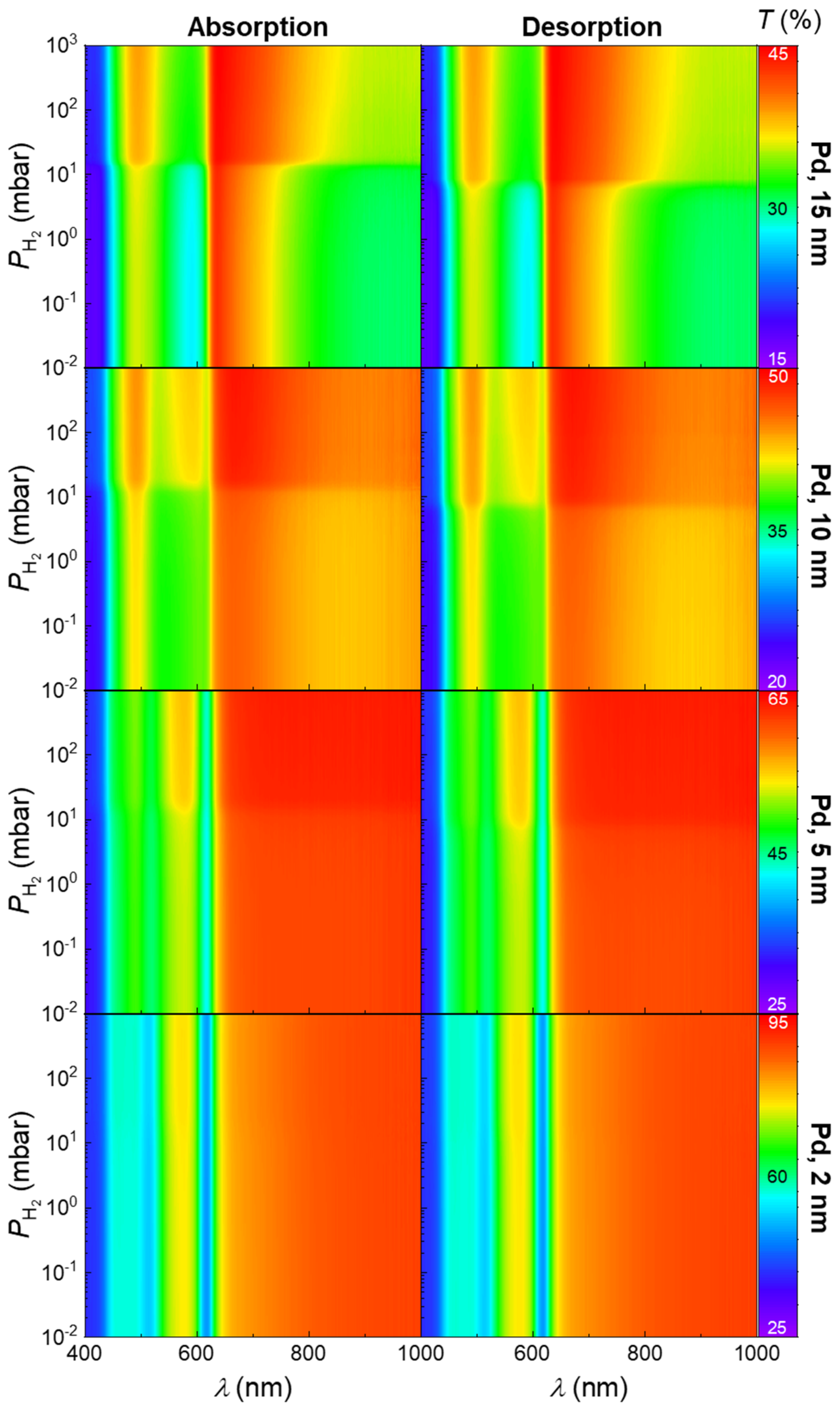

Supplementary Figure 23. Spectra response of Pd NP_t^{50} optical hydrogen sensor (t = 2, 5, 10, and 15 nm) to an increasing (left column)/decreasing (right column) hydrogen pressure.

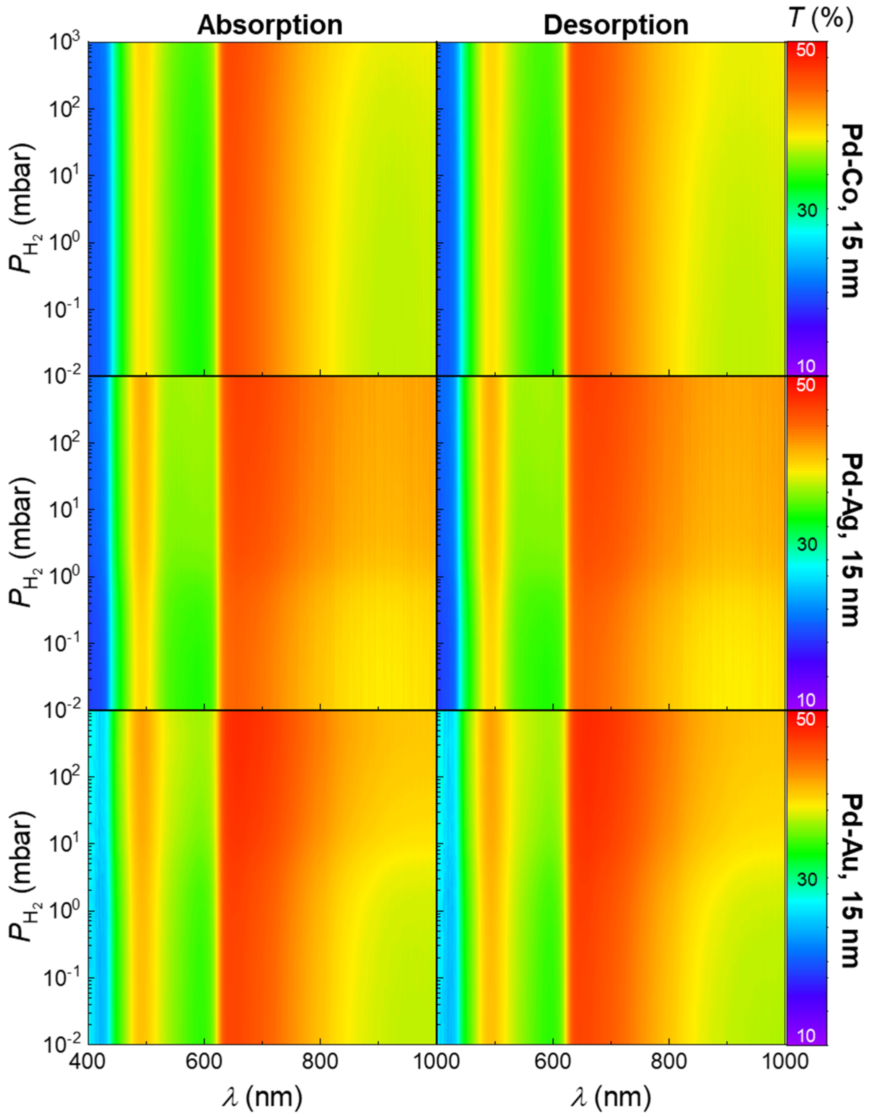

Supplementary Figure 24. Spectra response of NP_{15}^{50} optical hydrogen sensor (with different film composition) to an increasing (left column)/decreasing (right column) hydrogen pressure.

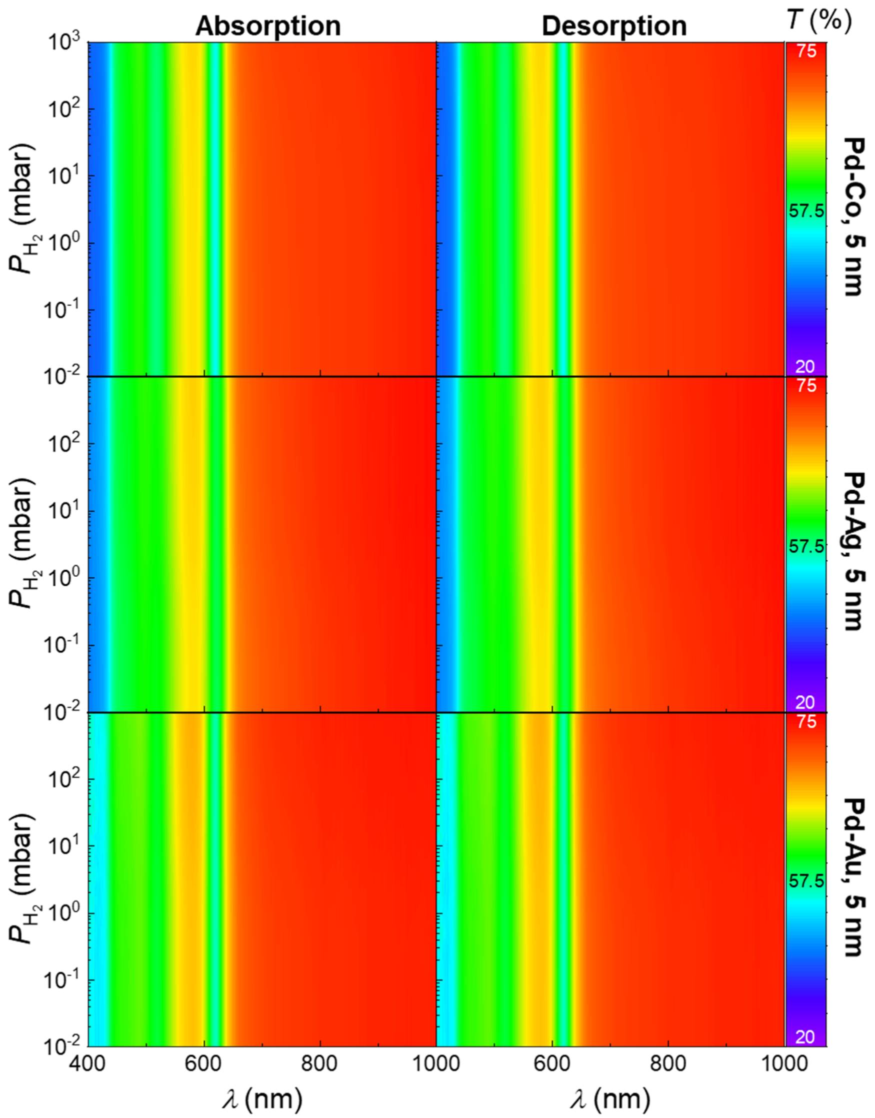

Supplementary Figure 25. Spectra response of NP_5^{50} optical hydrogen sensor (with different film composition) to an increasing (left column)/decreasing (right column) hydrogen pressure.

S9. Noise evaluation

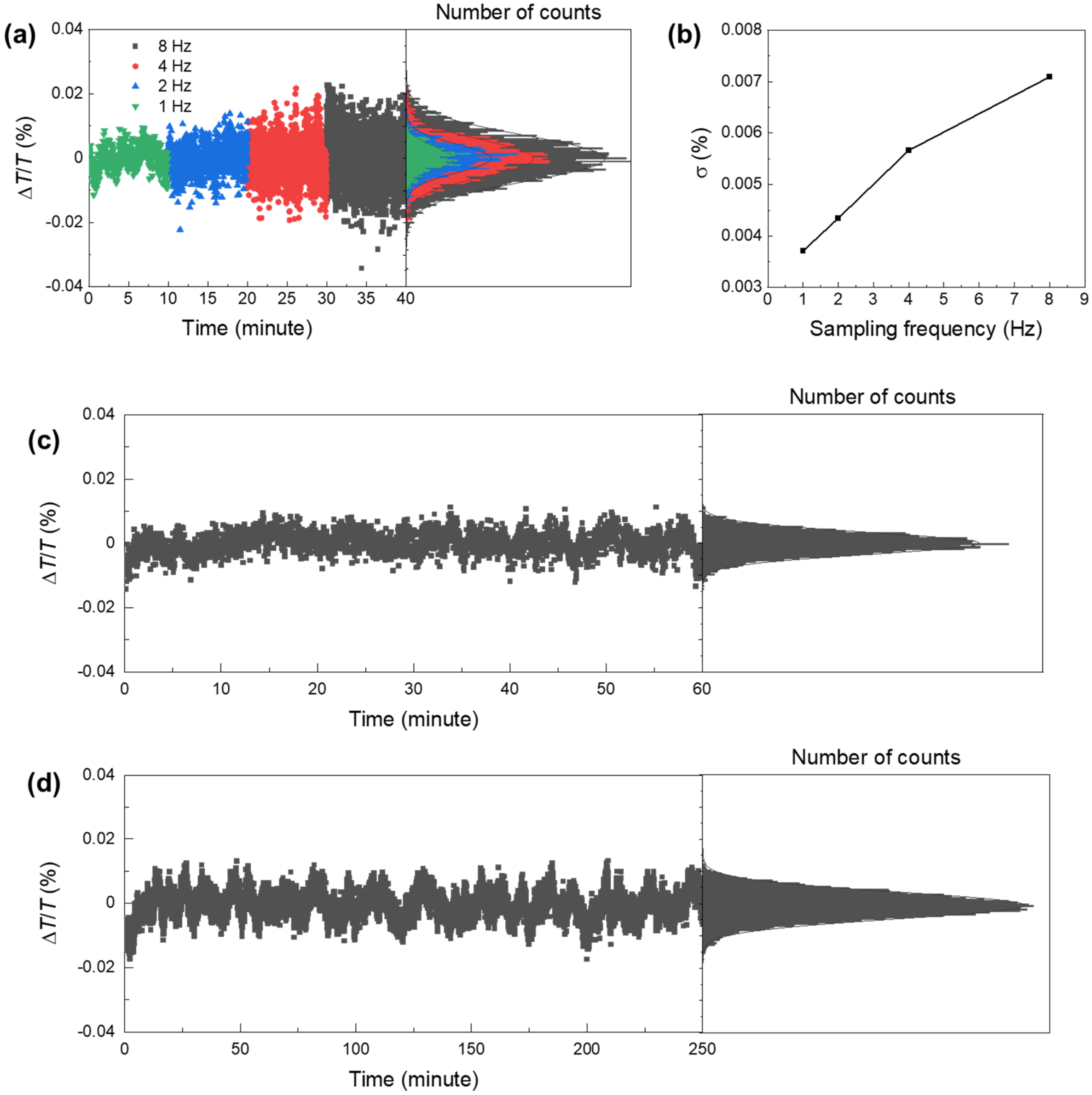

Supplementary Figure 26. (a) Experimental signal noise with different sampling frequencies of PdCo NP$_5^{50}$ sample, measured at 0 mbar of H_2 (left) and histogram plot of signal intensity (right). The distribution of noise exhibits normal distribution with standard deviations of σ. (b) The experimental signal noise, σ, as a function of sampling frequency. At 1 Hz of sampling frequency, σ = 0.0037 %. (c) Experimental signal noise at 1 Hz of sampling frequency of PdCo NP$_5^{50}$ sample, measured in flowing 4 % hydrogen in nitrogen (400 ml/min) (left), and histogram plot of signal intensity (right). At 1 Hz of

sampling frequency, σ = 0.0035 %. (d) Experimental signal noise at 1 Hz of sampling frequency of PdCo NP_5^{50} sample, measured in flowing 2 % hydrogen in synthetic air (400 ml/min) (left), and histogram plot of signal intensity (right). At 1 Hz of sampling frequency, σ = 0.0042 %.

S10. PdCo NP_5^{50} and PdCo NP_5^{50}/PMMA sensors stability

For a long-term stability assessment, we simply placed $Pd_{80}Co_{20}$ NP_5^{50} sample in the ambient condition (temperature 22-23°C, humidity 35% RH) and characterize the response time and limit of detection (LOD) of the sensor in a weekly basis (week 1 to 6). These obtained results are then compared to a 10-month-old sample, in Supplementary Fig. 27.

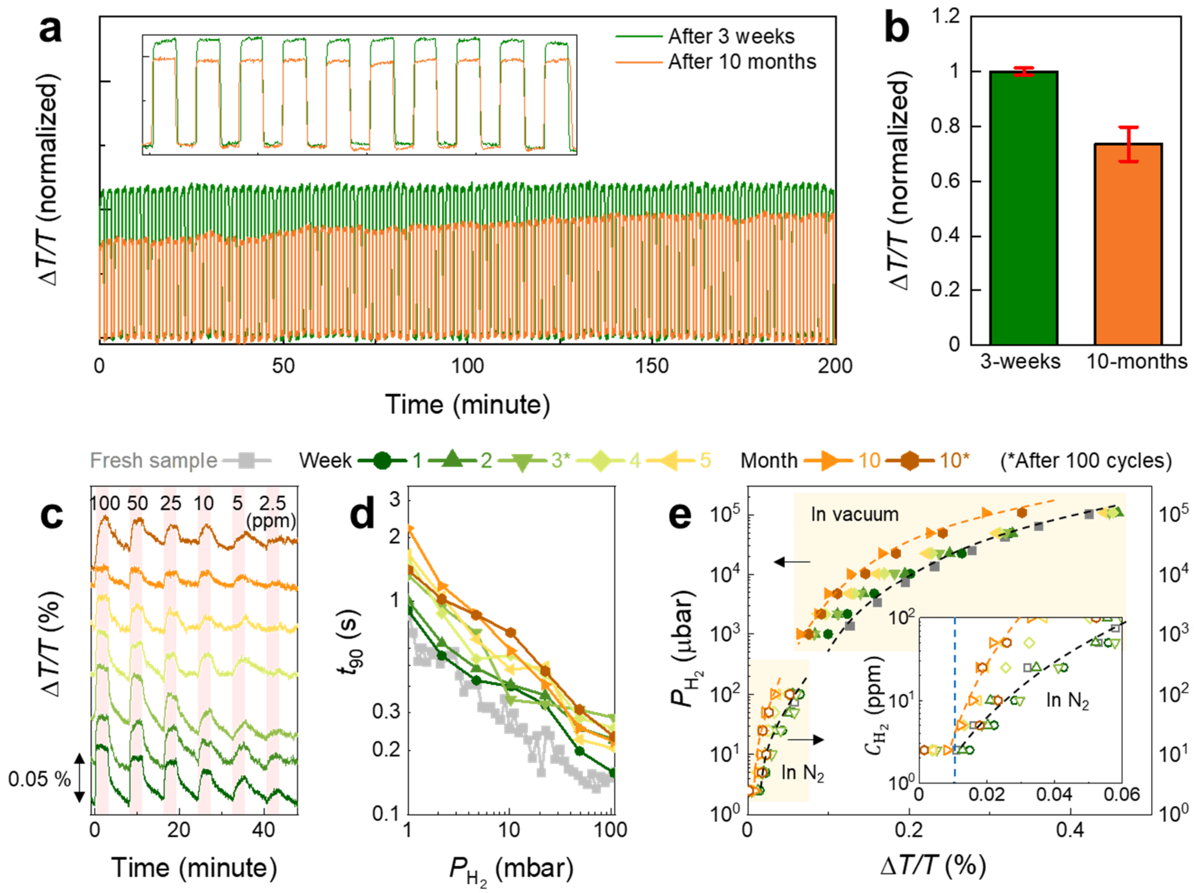

Supplementary Figure 27. (a) $\Delta T/T$ response of $Pd_{80}Co_{20}$ NP_5^{50} (3-weeks and 10-months old) upon 100 cycles (1/1 minute of loading/unloading) of 2% H_2 in synthetic gas (400 ml/min) and (b) $\Delta T/T$ response normalized to the response of a fresh sample, obtained in the same condition. The error bars denote the standard deviation from 100 cycles. (c) Long-term $\Delta T/T$ response of $Pd_{80}Co_{20}$ NP_5^{50} with different hydrogen concentrations (C_{H_2}) of 100 – 2.5 ppm, measured in flowing nitrogen (400 ml/min).

Shaded areas denote the periods where the sensor is exposed to hydrogen. Note that the $\Delta T/T$ response data presented in Fig. 5b and this figure (week 1) are identical. (d) Long-term response time of $Pd_{80}Co_{20}$ NP_5^{50} with 1-100 mbar pure hydrogen pulse. (e) Measured $\Delta T/T$ response as a function of P_{H_2} in vacuum/pure hydrogen (solid symbol) and C_{H_2} in flowing nitrogen (400 ml/min) (half-up filled symbols). Inset: the blue dashed line denotes the defined LOD at $3\sigma \approx 0.011$ % ($\sigma = 0.0035$ %, is the noise of the acquired signal with N_2 carrier air, see Supplementary Section 9).

The $Pd_{80}Co_{20}$ NP_5^{50} sample shows a good stability without the sign of degradation, upon >300 of (de)hydrogenation cycles. The first 100 cycles (1/1 minute of loading/unloading with 2% H_2 in synthetic gas) of 3-weeks old and 10-months old sample are summarized in Supplementary Fig. 27a. While a noticeable reduction of sensor signal due to aging can be seen in 10-months old sample (Supplementary Fig. 27b), upon cycling, the signal is recovering back to that of a fresh sample.

The sign of the aging effect can be seen by the degraded performances of the sensor over the times. In particular, the response time t_{90} (at $P_{H_2} = 1 - 100$ mbar) increases about ~2 times and ~3 times after a period of 4-weeks and 10-months, respectively (Supplementary Fig. 27c). In addition, the long-term $\Delta T/T$ responses show a significant reduction both in vacuum mode and flow mode (Supplementary Figs. 27d and e), just after 4-5 weeks in air. We ascribed the degradation of the sensor to a small trace amount of poison gases exist in the ambient air, (*e.g.* CO) (see the deactivation test with CH_4, CO_2, and CO in Figure 6). However, we note that the response time and LOD of the sample still are <2.5 s (at 1 mbar) and <10 ppm, respectively, over a >10-month period. The degradation of the $Pd_{80}Co_{20}$ NP_5^{50} sample upon long-term storage in air can affect to the accuracy, sensitivity, and response time of the sensor. Hence, we look for a solution to mitigate this process. Inspired by the work of Nugroho *et al.*,[18] we coated the $Pd_{80}Co_{20}$ NP_5^{50} sample with

a ~50-nm layer of polymethyl methacrylate (PMMA) (by spin-coating of PMMA dissolved in acetone, more details can be found in Methods section), which has been demonstrated to effectively block the poisonous species. In this test, we store the PMMA-coated and uncoated samples in an identical condition and measure their sensing metrics.

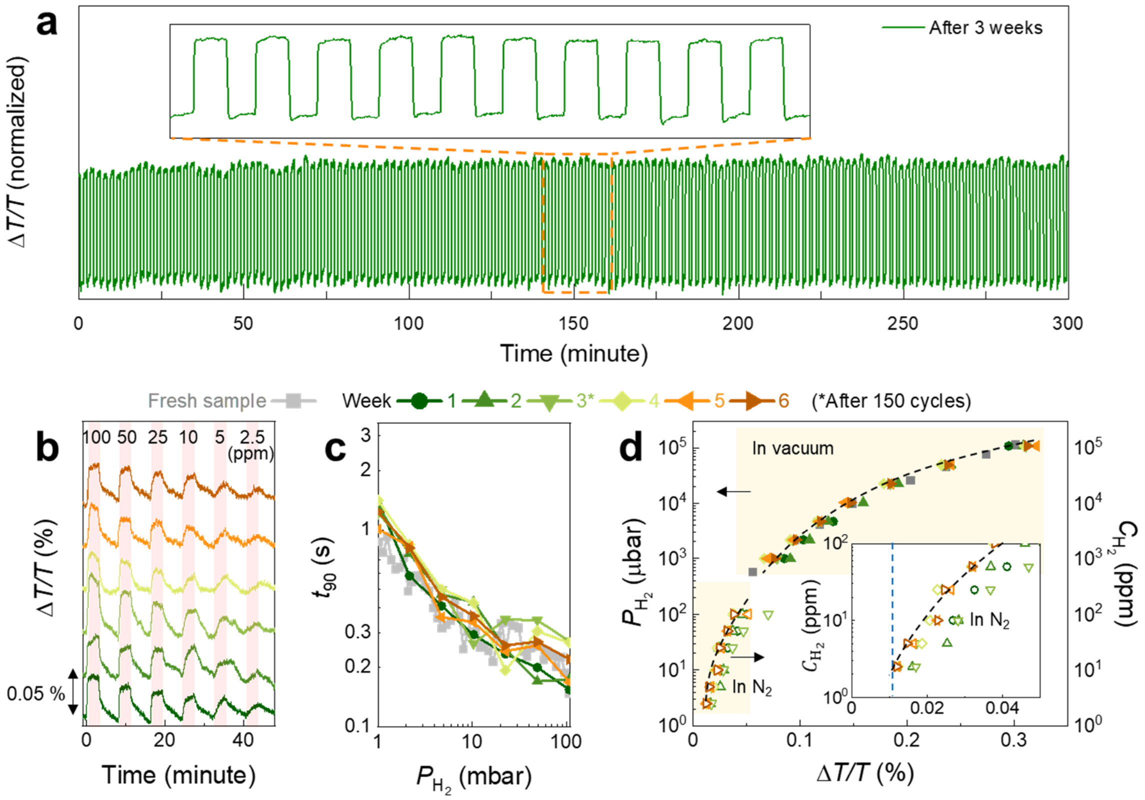

Supplementary Figure 28. (a) $\Delta T/T$ response of $Pd_{80}Co_{20}$ NP_5^{50}/PMMA (3-weeks old) upon 150 cycles (1/1 minute of loading/unloading) of 2% H_2 in synthetic gas (400 ml/min). (b) Long-term $\Delta T/T$ response of $Pd_{80}Co_{20}$ NP_5^{50}/PMMA with different hydrogen concentrations (C_{H_2}) of 100 – 2.5 ppm, measured in flowing nitrogen (400 ml/min). Shaded areas denote the periods where the sensor is exposed to hydrogen. (c) Long-term response time of $Pd_{80}Co_{20}$ NP_5^{50}/PMMA with 1-100 mbar pure hydrogen pulse. (d) Measured $\Delta T/T$ response as a function of P_{H_2} in vacuum/pure hydrogen (solid symbol) and with different C_{H_2} in flowing N_2 (half-up filled symbols). Inset: the blue dashed line denotes the defined LOD at 3σ ≈ 0.011 % (σ = 0.0035 %, is the noise of the acquired signal with N_2 carrier air, see Supplementary Section 9).

The sensing performances of $Pd_{80}Co_{20}$ NP_5^{50}/PMMA sensor are summarized in Supplementary Fig. 28. After storing in air for 6-weeks and underwent >200 cycles of (de)hydrogenation with 2% H_2, we observe very little variances in response time over the pressure range of 1-100 mbar (t_{90} are <1.5 s (at 1 mbar)) and insignificant reduction of sensor signal upon exposure to pulses of very low H_2 concentration (100 – 2.5 ppm). Clearly, the degradation of the sensor performance is significantly slowed-down with a polymer coating layer.

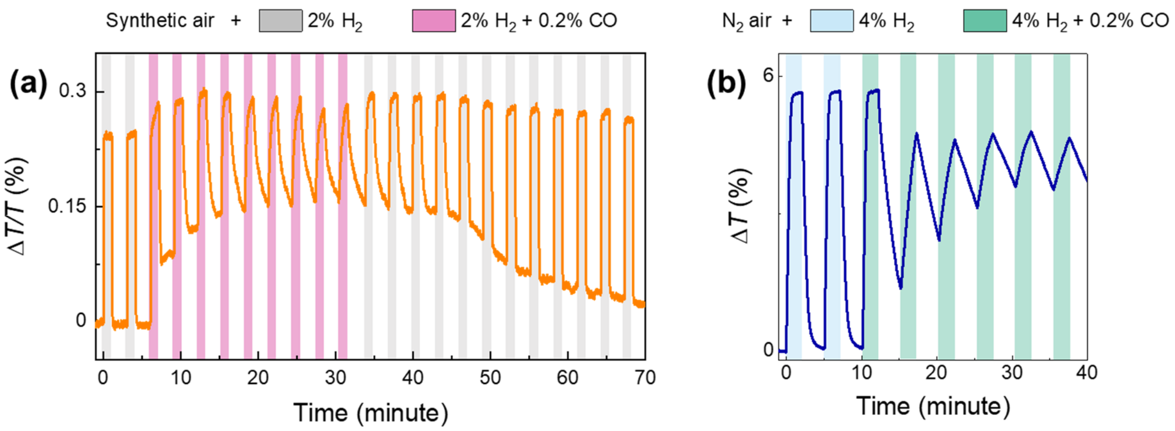

Supplementary Figure 29. (a) Time-resolved $\Delta T/T$ response of $Pd_{80}Co_{20}$ NP_5^{50} (to 2 pulses of 2% H_2 followed by 9 pulses of 2% H_2 + 0.2% CO, and 12 pulses of 2% H_2, with synthetic air as a carrier gas), shows that sensor signal and slow response/release time of a poisoned sensor can be recovered upon several (de)hydrogenation cycles. Note that the $\Delta T/T$ responses in this figure and Figure 6a are identical. (b) Time-resolved $\Delta T/T$ response of Pd NP_{15}^{50} (to 2 pulses of 4% H_2 followed by 6 pulses of 4% H_2 + 0.2% CO, with nitrogen air as a carrier gas) for comparison purposes.

S11. Sensing performances of a fresh PdCo NP_5^{50}/PMMA sensors

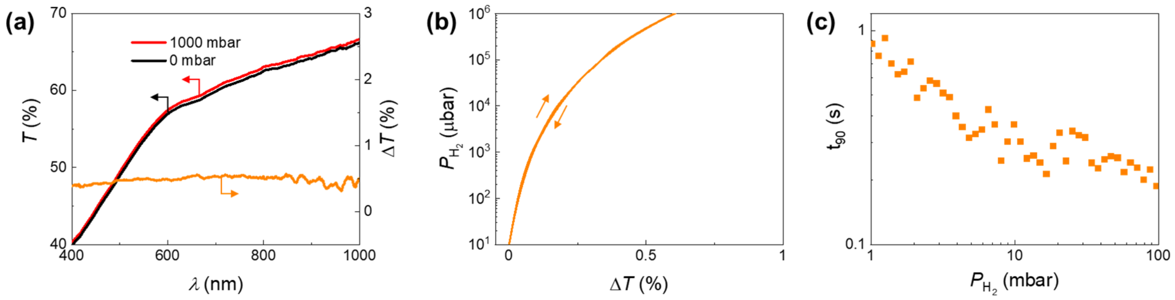

Supplementary Figure 30. (a) Experimental optical transmission spectra $T(\lambda)$ at P_{H_2} = 0 and 1000 mbar, and optical transmission changes $\Delta T(\lambda) = T_{1000\ mbar}$ - $T_{0\ mbar}$ of PdCo NP_5^{50}/PMMA sensor. (b) Optical hydrogen sorption isotherm extracted at $\Delta T(\lambda)$ maxima. Arrows denote the sorption direction. (c) Response time of sensors with pulse of hydrogen pressure from 1 mbar to 100 mbar.

S12. Sensing metrics of state-of-art optical hydrogen sensor (at room-temperature)

**Sensing platform**		***t*₉₀ (s) (@ 40 mbar)**	***t*₉₀ (s) (@ 1 mbar)**	**LOD (ppm)**	**Hysteresis-free?**	**Ref.**
$Pd_{80}Co_{20}$ NP		≤ 0.15	0.85	2.5	Yes	This work
PdAu nano-particles @PTFE@PMMA	190 × 25 (nm^2)	0.3	> 2	10<br>3 (by extrapolation)	Yes	[18]
	100 × 25 (nm^2)	< 0.3	1	n.a.*<br>< 1000 (estimated)		
Pd nano-disk array		> 10	-	50	n.a.	[19]
Pd bilayer lattices		~ 900	55	-	n.a.	[14]
PdAuCu nano-particles		0.4	-	5	Yes	[20]
PdAu nanostructures		40	-	-	Yes	[21]
Pd strip		-	20	10	n.a.	[22]
PdY film		6	-	1000	n.a.	[23]
Pd/SiO2/Au		3	-	5000	n.a.	[24]
Pd/Au film		4.5	-	-	n.a.	[25]

Supplementary Table 1. Sensing metrics of state-of-art optical hydrogen sensor (at room-temperature). *not addressed.

Supplementary References

1 Ingram, W. M., Han, C., Zhang, Q. & Zhao, Y. Optimization of Ag-coated polystyrene nanosphere substrates for quantitative surface-enhanced Raman spectroscopy analysis. *J. Phys. Chem. C* **119**, 27639-27648 (2015).

2 Larsen, G. K., He, Y., Ingram, W. & Zhao, Y. Hidden chirality in superficially racemic patchy silver films. *Nano Lett.* **13**, 6228-6232 (2013).

3 Sykes, M. F. & Essam, J. W. Exact critical percolation probabilities for site and bond problems in two dimensions. *Journal of Mathematical Physics* **5**, 1117-1127 (1964).

4 Griessen, R., Strohfeldt, N. & Giessen, H. Thermodynamics of the hybrid interaction of hydrogen with palladium nanoparticles. *Nature materials* **15**, 311-317 (2016).

5 Sachs, C. *et al.* Solubility of hydrogen in single-sized palladium clusters. *Phys. Rev. B* **64**, 075408 (2001).

6 Schwarz, R. & Khachaturyan, A. Thermodynamics of open two-phase systems with coherent interfaces. *Phys. Rev. Lett.* **74**, 2523 (1995).

7 Wadell, C. *et al.* Thermodynamics of hydride formation and decomposition in supported sub-10 nm Pd nanoparticles of different sizes. *Chemical Physics Letters* **603**, 75-81 (2014).

8 Li, G. *et al.* Hydrogen storage in Pd nanocrystals covered with a metal–organic framework. *Nature materials* **13**, 802-806 (2014).

9 Langhammer, C., Zorić, I., Kasemo, B. & Clemens, B. M. Hydrogen storage in Pd nanodisks characterized with a novel nanoplasmonic sensing scheme. *Nano Lett.* **7**, 3122-3127 (2007).

10 Frazier, G. & Glosser, R. Phase diagrams of thin films of the palladium hydrogen system using a quartz crystal thickness monitor. *J. Phys. D: Appl. Phys.* **12**, L113 (1979).

11 Zabel, H. & Peisl, H. Sample-shape-dependent phase transition of hydrogen in niobium. *Phys. Rev. Lett.* **42**, 511 (1979).

12 Pivak, Y., Schreuders, H., Slaman, M., Griessen, R. & Dam, B. Thermodynamics, stress release and hysteresis behavior in highly adhesive Pd–H films. *international journal of hydrogen energy* **36**, 4056-4067 (2011).

13 Zhdanov, V. P. & Kasemo, B. Kinetics of the formation of a new phase in nanoparticles. *Chemical Physics Letters* **460**, 158-161 (2008).

14 Luong, H. M. *et al.* Bilayer Plasmonic Nano-lattices for Tunable Hydrogen Sensing Platform. *Nano Energy* **71**, 104558 (2020).

15 Li, Y. *et al.* Surface plasmon coupling enhanced dielectric environment sensitivity in a quasi-three-dimensional metallic nanohole array. *Opt. Express* **18**, 3546-3555 (2010).

16 Wadell, C. *et al.* Hysteresis-free nanoplasmonic Pd–Au alloy hydrogen sensors. *Nano Lett.* **15**, 3563-3570 (2015).

17 Makrides, A. Absorption of Hydrogen by Silver—Palladium Alloys. *The Journal of Physical Chemistry* **68**, 2160-2169 (1964).

18 Nugroho, F. A. *et al.* Metal–polymer hybrid nanomaterials for plasmonic ultrafast hydrogen detection. *Nature materials* **18**, 489 (2019).

19 Herkert, E., Sterl, F., Strohfeldt, N., Walter, R. & Giessen, H. Low-Cost Hydrogen Sensor in the ppm Range with Purely Optical Readout. *ACS sensors* **5**, 978-983 (2020).

20 Darmadi, I., Nugroho, F. A. A., Kadkhodazadeh, S., Wagner, J. B. & Langhammer, C. Rationally-Designed PdAuCu Ternary Alloy Nanoparticles for Intrinsically Deactivation-

Resistant Ultrafast Plasmonic Hydrogen Sensing. *ACS sensors* **4**, 1424-1432, doi:10.1021/acssensors.9b00610 (2019).
21 Nugroho, F. A. A., Eklund, R., Nilsson, S. & Langhammer, C. A fiber-optic nanoplasmonic hydrogen sensor via pattern-transfer of nanofabricated PdAu alloy nanostructures. *Nanoscale* **10**, 20533-20539 (2018).
22 He, J. *et al.* Integrating plasmonic nanostructures with natural photonic architectures in Pd-modified Morpho butterfly wings for sensitive hydrogen gas sensing. *RSC Adv.* **8**, 32395-32400 (2018).
23 Song, H. *et al.* Optical fiber hydrogen sensor based on an annealing-stimulated Pd–Y thin film. *Sens. Actuator B-Chem.* **216**, 11-16 (2015).
24 Perrotton, C. *et al.* A reliable, sensitive and fast optical fiber hydrogen sensor based on surface plasmon resonance. *Opt. Express* **21**, 382-390 (2013).
25 Monzón-Hernández, D., Luna-Moreno, D. & Martínez-Escobar, D. Fast response fiber optic hydrogen sensor based on palladium and gold nano-layers. *Sens. Actuator B-Chem.* **136**, 562-566 (2009).
